# ROS-scavenging nanoparticles loaded with tectorigenin protect against acetaminophen-induced hepatotoxicity by interrupting the calcium/ROS-mediated pathogenic endoplasmic reticulum–Mitochondrial signaling cascade

**DOI:** 10.1016/j.bioactmat.2025.12.016

**Published:** 2025-12-13

**Authors:** Yaqi Zhang, Zeyuan Jin, Lvwan Xu, Zilong Zhong, Xinyu Wang, Changyou Gao, Lanjuan Li

**Affiliations:** a State Key Laboratory for Diagnosis and Treatment of Infectious Diseases, National Clinical Research Center for Infectious Diseases, China-Singapore Belt and Road Joint Laboratory on Infection Research and Drug Development, National Medical Center for Infectious Diseases, Collaborative Innovation Center for Diagnosis and Treatment of Infectious Diseases, The First Affiliated Hospital, Zhejiang University School of Medicine, Hangzhou, 310003, China; b Yuhang Institute of Medical Science Innovation and Transformation, Hangzhou, 311112, China; c MOE Key Laboratory of Macromolecular Synthesis and Functionalization, Department of Polymer Science and Engineering, Zhejiang University, Hangzhou, 310058, China; d Zhejiang Engineering Research Center for Interface Technology of Medical Polymers and Devices, Shaoxing Key Laboratory of Healthcare Materials and Application Technology, and Center for Healthcare Materials, Shaoxing Institute, Zhejiang University, Shaoxing, 312099, China; e The State Key Laboratory of Transvascular Implantation Devices, Zhejiang University, Hangzhou, 310009, China; f Dr. Li Dak Sum & Yip Yio Chin Center for Stem Cell and Regenerative Medicine, Zhejiang University, Hangzhou, 310058, China

**Keywords:** Acute liver injury, Reactive oxygen species, Endoplasmic reticulum stress, Mitochondrial dysfunction, Tectorigenin

## Abstract

Acetaminophen (APAP) overdose is a leading cause of acute liver injury (ALI) and acute liver failure (ALF) worldwide, representing a major clinical and public health challenge due to its rapid onset and high morbidity. Current clinical treatment is limited to N-acetylcysteine (NAC), but its efficacy is highly time-dependent and the prolonged regimen imposes additional clinical burdens and side effects. Natural compounds hold tremendous promise for hepatoprotection, but their clinical translation is limited by unfavorable physicochemical and pharmacokinetic properties. In this study, tectorigenin (Tec), an isoflavone possessing anti-inflammatory and antioxidative activity, was encapsulated within a reactive oxygen species (ROS)-responsive nanoplatform (PBHB@Tec) to enhance bioavailability and enable site-selective hepatoprotection. PBHB@Tec possessed diameters compatible with passage through hepatic sinusoidal fenestrae into the space of Disse enabling direct hepatocyte interaction, while exhibiting potent ROS scavenging activity and undergoing ROS-triggered morphological degradation that accelerated Tec release under oxidative conditions. In an APAP-induced ALI mouse model, PBHB@Tec markedly attenuated ALI phenotypes. Mechanistically, PBHB@Tec reduced endoplasmic reticulum (ER) stress, which alleviated ER Ca^2+^ leak and subsequently prevented mitochondrial Ca^2+^ overload. This, in turn, lowered mitochondrial ROS production and restored antioxidant defenses, collectively disrupting the feedforward calcium/ROS apoptotic cascade. These broad improvements in ER-mitochondrial homeostasis positioning PBHB@Tec as a promising ROS-responsive nanotherapy for APAP-induced hepatotoxicity.

## Highlights

• Microenvironment-adaptive nanocarrier enables targeted delivery and enhances therapeutic efficacy of poorly soluble flavonoids in acute liver injury.
• The nanoparticle system synergistically scavenges ROS and inhibits ER stress-mediated calcium overload in hepatocytes.
• Carrier–drug cooperation restores ER and mitochondrial redox balance, reducing hepatocyte apoptosis and liver injury.

## 1 Introduction

Drug-induced liver injury (DILI) is a severe adverse reaction to medications and other xenobiotics, manifesting either predictably after exposure to toxic doses of specific substances or unpredictably with many commonly used drugs [1]. The risk of DILI to cause acute liver injury (ALI) or even acute liver failure (ALF) presents a significant global public health concern. Based on the pattern of liver injury, hepatocellular DILI accounts for over half of all cases [[2], [3], [4]]. Acetaminophen (APAP), a widely used analgesic and antipyretic, is a leading cause of ALF worldwide following overdose [5]. Its hepatotoxicity is characterized by centrilobular hepatocyte necrosis [6]. The only therapeutic option approved by the US Food and Drug Administration (FDA) for APAP overdose is N-acetylcysteine (NAC). However, its clinical application is hindered by high dosing requirements, prolonged treatment duration due to low bioavailability, and associated adverse reactions [7].

A central yet underexplored mechanism of APAP-induced liver injury (AILI) lies in the interplay between oxidative stress and organelle crosstalk. Following APAP overdose, cytochrome P450-mediated metabolism generates excessive reactive oxygen species (ROS), triggering profound mitochondrial oxidative stress—now recognized as a key driver of hepatotoxicity [8]. Mitochondria and the endoplasmic reticulum (ER) networks are closely linked both functionally and physically [9]. Under ROS burden, redox-sensitive ER calcium channels, such as inositol 1,4,5-trisphosphate receptor (IP3R) and the ryanodine receptor (RyR), become dysregulated, promoting pathological calcium flux. The resulting cytosolic calcium surge is taken up by mitochondria via voltage-dependent anion channels (VDAC) and the mitochondrial calcium uniporter (MCU). This calcium overload stimulates the mitochondrial respiratory chain, producing even more ROS, which in turn feeds back to the ER and further impairs calcium channel function. Critically, this sets up a self-perpetuating ROS–Ca^2+^ pathological loop: escalating oxidative stress and calcium dysregulation provoke sustained ER stress, defective protein folding, and activation of the unfolded protein response (UPR) [10], orchestrated by stress sensors including inositol-requiring enzyme 1 (IRE1), activating transcription factor 6 (ATF6), and protein kinase R-like ER kinase (PERK). If stress is sustained, the PERK–eukaryotic translation initiation factor 2α (eIF2α)–activating transcription factor 4 (ATF4)–C/EBP-homologous protein (CHOP) signaling axis drives apoptotic gene expression, tipping the balance toward cell death [11]. Simultaneously, oxidative protein folding in the ER by enzymes such as endoplasmic reticulum oxidoreductin-1 (ERO1α) contributes additional ROS, compounding the redox imbalance. As ROS and calcium signaling mutually reinforce each other, persistent disruption of ER and mitochondrial homeostasis leads to mitochondrial permeability transition pore (mPTP) opening, release of pro-apoptotic factors, and irreversible cell injury [12,13]. Thus, the ER–mitochondria crosstalk driven by the ROS–Ca^2+^ feedforward loop is now recognized as a core mechanism linking APAP-induced oxidative stress to hepatocyte apoptosis.

While natural antioxidants have long been investigated as adjunct therapies for AILI, their clinical translation remains limited by chemical instability and poor bioavailability [[14], [15], [16]]. Tectorigenin (Tec, PubChem CID: 5281811), an isoflavone derived from the traditional Chinese herb Belamcandae Rhizoma [17], possesses potent antioxidant and anti-inflammatory properties and shows hepatoprotective promise in preclinical studies [[18], [19], [20]]. However, like many other natural compounds, Tec faces significant biopharmaceutical challenges: it is classified as a Class IV compound in the Biopharmaceutical Classification System (BCS), indicating both low solubility and low permeability. Such pharmacokinetic limitations often require pre-treatment strategies to achieve sufficient therapeutic efficacy, yet this approach is clinically impractical given that DILI typically arises acutely and unpredictably.

Nanotechnology-based drug delivery systems are a common strategy to improve the solubility and bioavailability of hydrophobic drugs [21]. However, the functional capabilities of conventional passive nanocarriers are mostly limited to enhanced delivery and accumulation. Such passive systems are increasingly insufficient to address complex and self-amplifying pathologies like AILI, which involve dynamic, multi-organelle feedback loops—particularly the ER–mitochondria axis. In contrast, active nanoplatforms that both respond to and remodel the pathological hepatic microenvironment could deliver fundamentally superior clinical benefit. Among such strategies, ROS-scavenging nanomaterials are especially attractive, as they directly modulate the redox microenvironment, mitigate oxidative stress, and interrupt the vicious cycle of cellular injury in various disease models [[22], [23], [24]]. Despite this promise, the development of truly effective ROS-responsive systems for liver disease therapy remains challenging. In the expanding landscape of ROS-responsive nanomaterials for hepatic applications, several systems based on motifs such as thioketal, boronic ester, or selenium/tellurium-containing polymers have been explored [25,26]. However, these materials often suffer from limitations such as synthetic complexity, potential toxicity of degradation byproducts, or suboptimal ROS-scavenging efficiency, collectively restricting further clinical advancement.

**Scheme 1:**
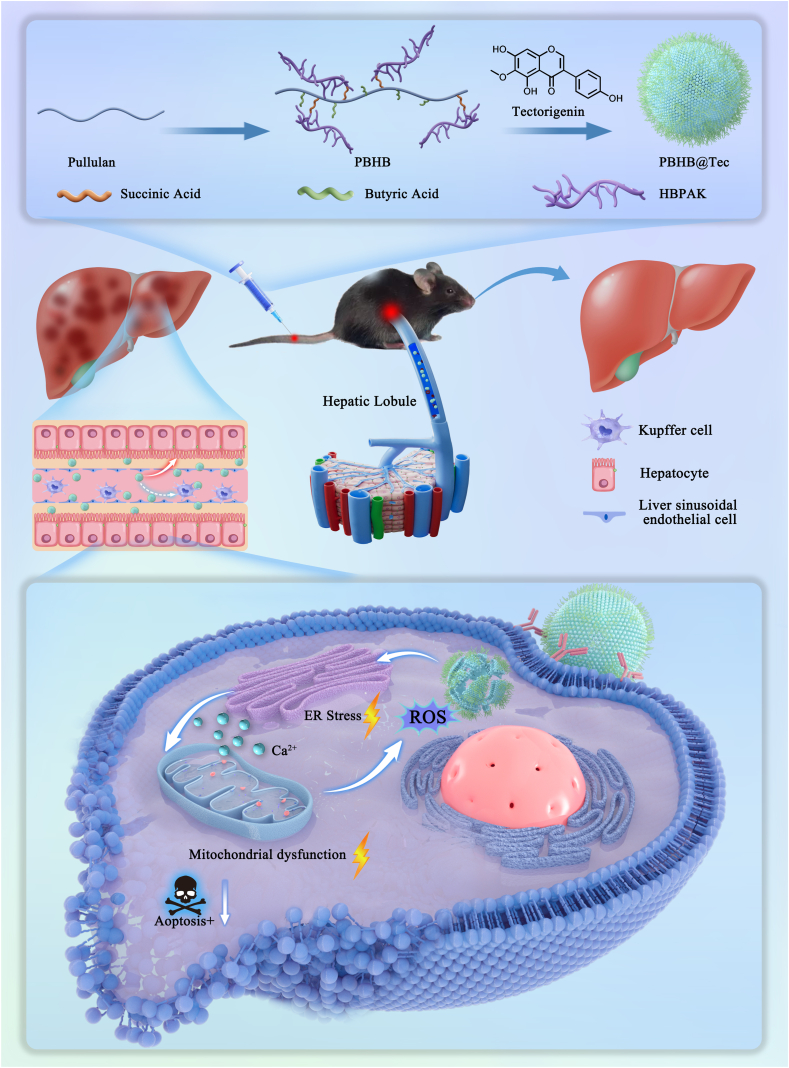
Schematic illustration of the *in vivo* therapy strategy for acetaminophen-induced acute liver injury using ROS-scavenging nanoparticles loaded with Tec. The intravenously administered PBHB@Tec nanoparticles, with an engineered polymeric structure, preferentially accumulate in damaged hepatocytes. Upon sensing elevated ROS levels in the injured liver, HBPAK collaborates with the released Tec to alleviate ER stress and mitochondrial dysfunction, thereby suppressing hepatocyte apoptosis and promoting liver repair.

In this study, we synthesized a conjugate of butyrylated pullulan and hyperbranched polyaminoketal (HBPAK) and formulated it into nanoparticles for targeted delivery of Tec to injured hepatocytes. The design leverages pullulan—a biodegradable polysaccharide [27], classified as Generally Recognized as Safe (GRAS) by the FDA [28], with intrinsic hepatocyte-targeting ability mediated by the asialoglycoprotein receptor (ASGPR) [[29], [30], [31]]. To further enhance hepatocyte targeting and reduce capture by non-parenchymal cells, the nanoparticles were engineered to be sufficiently small to facilitate transit through the fenestrated liver sinusoidal endothelium into the space of Disse, where hepatocytes reside [32]. Butyrylation and HBPAK conjugation endow the platform with hydrophobic drug encapsulation, ROS scavenging activity, and ROS-triggered nanoparticle disassembly. The ROS-scavenging function of HBPAK actively reduces the intracellular redox burden while facilitating rapid release of encapsulated Tec, maximizing therapeutic action at the site of injury. This integrated approach disrupts the positive-feedback ROS–Ca^2+^ amplification loop in the ER–mitochondria circuit, restoring organelle homeostasis and preventing hepatocyte loss. By integrating a smart stimulus-responsive nanoplatform with the hepatoprotective efficacy of Tec, this strategy provides a rational approach to achieve targeted intervention in the ER–mitochondria stress axis, a central pathway underlying APAP-induced hepatotoxicity (Scheme 1).

## 2 Results and discussion

### 2.1 Preparation and physicochemical characterization of PBHB@Tec

**Fig. 1:**
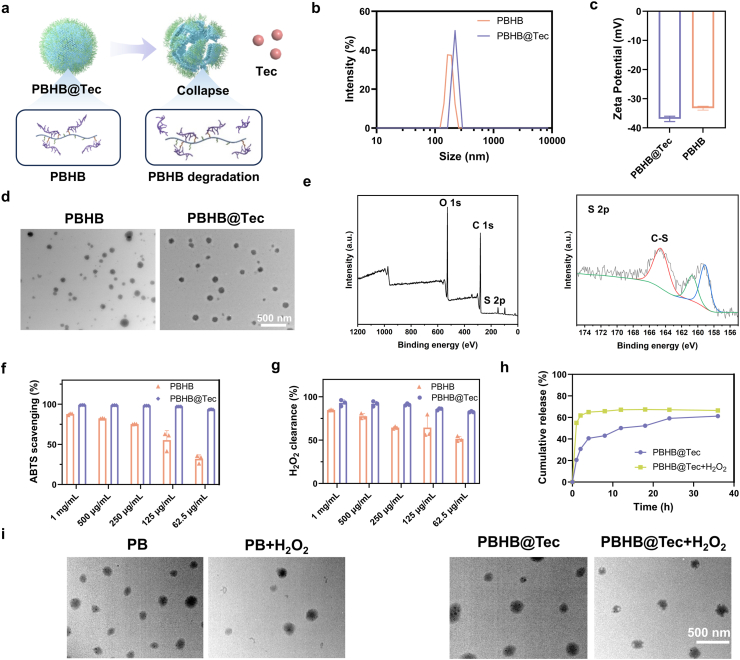
Characterization and ROS-responsiveness of PBHB@Tec. (a) Schematic illustration of the preparation of PBHB@Tec nanoparticles and their ROS-triggered degradation as well as Tec release. (b) DLS analysis showing the size distribution of PBHB and PBHB@Tec nanoparticles. (c) Zeta potential measurements of PBHB and PBHB@Tec. (d) TEM images of PBHB and PBHB@Tec nanoparticles. Scale bar: 500 nm. (e) XPS spectra indicating the chemical composition of PBHB@Tec. (f, g) *In vitro* ABTS radical scavenging activity and H_2_O_2_ clearance ability of PBHB and PBHB@Tec at various concentrations. (h) Cumulative release profiles of Tec from PBHB@Tec nanoparticles in the absence and presence of H_2_O_2_. (i) TEM images of PB, PB incubated with H_2_O_2_, PBHB@Tec, and PBHB@Tec incubated with H_2_O_2_ to visualize nanoparticle stability and ROS-triggered degradation. Scale bar: 500 nm. Data are expressed as mean ± SD (n = 3).

Initially, we designed a conjugate of butyrylated pullulan and HBPAK, termed PBHB (Supplementary Information, Scheme 1). HBPAK is an antioxidant polymer [33], and its conjugation with butyrylated pullulan enables the resulting construct to simultaneously target injured hepatocytes and exert antioxidant effects. To this end, we screened intermediates with different grafting degrees and successfully synthesized PBHB (Fig. S1 and Fig. S2). By optimizing the concentration of the surfactant polyvinyl alcohol (PVA) and the solid content in the organic phase, we obtained blank nanoparticles and Tec-loaded nanoparticles with optimized particle sizes (Fig. S3 and Fig. S4). The drug-loaded particles exhibited a slightly larger diameter (Fig. 1b). All particles carried a negative surface charge (Fig. 1c). Transmission electron microscopy (TEM) results further confirmed the uniform size distribution of the nanoparticles (Fig. 1d). The resulting PBHB@Tec nanoparticles, with a peak size of approximately 200 nm, fall well within the permissive 50–300 nm pore size range of the fenestrated liver sinusoidal endothelium [34,35]. This size allows the particles to readily enter the space of Disse and access hepatocytes. X-ray photoelectron spectroscopy (XPS) analysis of the nanoparticle powder showed a characteristic C–S peak at 164.5 eV (Fig. 1e). This result, together with the results of ^1^H NMR and N 1s spectrum (Figs. S2 and S5), collectively confirmed the successful formation of nanoparticles by the PB-HBPAK conjugate, PBHB.

To achieve targeted treatment of injured hepatocytes during ALI, the nanoparticles must overcome the barrier of the sinusoidal endothelium and release Tec precisely in hepatocytes under high ROS conditions. The hydrated particle size in the nanometer range allows efficient penetration through the sinusoidal fenestrae. Moreover, conjugation with HBPAK enables ROS-triggered acceleration of backbone degradation, leading to subsequent nanoparticle disintegration. At a concentration of 62.5 μg/mL, PBHB@Tec exhibited robust antioxidant activity, with scavenging rates of 93.72 % and 83.42 % against 2,2′-Azino-bis(3-ethylbenzothiazoline-6-sulfonic acid) (ABTS) and H_2_O_2_, respectively. Meanwhile, blank nanoparticles (PBHB) without Tec showed lower scavenging capacity at the same concentration (Fig. 1f and g). The antioxidant activity of PBHB@Tec was stronger than that of PBHB due to the inherent antioxidant properties of Tec itself. This property also allows sensitive response to H_2_O_2_ in inflammatory milieus, leading to rapid release of Tec within 4 h under stimulation, with a cumulative release slightly higher than that of the group without H_2_O_2_ treatment (Fig. 1h). TEM images revealed that, after H_2_O_2_ exposure, non-ROS-responsive PB nanoparticles maintained their morphology, whereas PBHB@Tec showed surface roughening, structural defects, and a reduction in size (Fig. 1i). These findings further support that, under inflammatory ROS conditions, the nanoparticles undergo structural erosion and disruption, facilitating accelerated release of Tec and concomitant mitigation of oxidative stress.

Collectively, these characterization results and functional assessments underscore the utility of PBHB@Tec as an intelligent drug delivery platform. The novelty and advantage of PBHB@Tec are multi-fold. First, in terms of synthetic simplicity and biocompatibility, PBHB is fabricated from starting materials that are either composed of or derived from FDA-validated, highly biocompatible building blocks, enabling a facile conjugation process and enhancing its translational potential. This nanoparticle system readily encapsulates Tec with high efficiency, improving Tec's aqueous dispersibility and physical stability. Second, PBHB@Tec combines robust colloidal stability with a unique ROS-responsive architecture. The HBPAK core, rich in thioketal bonds, serves a dual function: it acts as a highly efficient ROS scavenger to mitigate oxidative stress directly, and undergoes cleavage in the high-ROS microenvironment, facilitating the on-demand disintegration of the nanoparticle and rapid release of Tec. Therefore, PBHB@Tec stands out by harmonizing synthetic accessibility, inherent biocompatibility, potent antioxidant activity, and a smart, dual-function response mechanism, establishing it as a promising intelligent platform with integrated targeting, antioxidant, and stimulus-responsive capabilities.

### 2.2 PBHB@Tec ameliorates AILI in mice

**Fig. 2:**
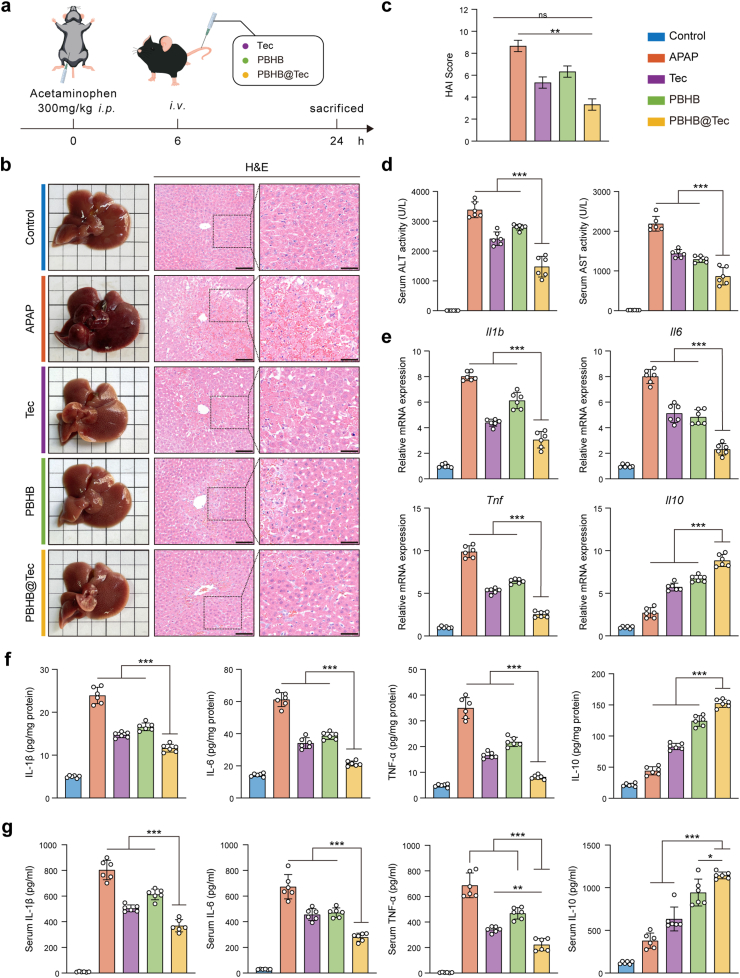
*In vivo* therapeutic effects of PBHB@Tec in the APAP-induced ALI mouse model. (a) Schematic overview of the animal experiment design. (b) Representative gross liver photographs and H&E staining images showing histopathological effects. Scale bars: 100 μm (20×), 20 μm (40×). (c) HAI quantifying liver injury severity. (d) Serum ALT and AST levels as markers of liver function. (e) Hepatic mRNA expression levels of *Il1b*, *Il6*, *Tnf*, and *Il10*. (f) Hepatic protein levels of IL-1β, IL-6, TNF-α, and IL-10. (g) Serum protein levels of IL-1β, IL-6, TNF-α, and IL-10. Data are expressed as mean ± SD (n = 6). ∗*p* < 0.05; ∗∗*p* < 0.01; ∗∗∗*p* < 0.001; ns, not significant.

To evaluate the therapeutic potential of PBHB@Tec *in vivo*, mice were administered 300 mg/kg APAP via intraperitoneal injection to induce ALI. As illustrated in the experimental timeline (Fig. 2a), mice received an intravenous injection of Tec, PBHB, PBHB@Tec or saline at 6 h after APAP administration. Samples were collected at 24 h post-APAP injection for subsequent analyses. Compared to the enlarged, darkened, and mottled appearance of the APAP-injured liver, the livers of mice treated with PBHB@Tec visibly appeared a brighter red color and a softer texture, closely resembling those of the control group. Histopathological analysis of liver tissues revealed that APAP administration induced substantial hepatocellular necrosis and disruption of normal hepatic architecture. Treatment with Tec or PBHB alone could alleviate hepatocellular damage; however, the PBHB@Tec-treated group demonstrated greater improvement, exhibiting markedly reduced necrosis and largely restored hepatic cellular morphology (Fig. 2b). The differences in histological activity index (HAI) further quantified these changes (Fig. 2c). These histopathological findings were further corroborated by biochemical analyses assessing liver function. Serum levels of the key liver injury biomarkers alanine aminotransferase (ALT) and aspartate aminotransferase (AST) were significantly reduced in the PBHB@Tec-treated group, highlighting its superior hepatoprotective potential (Fig. 2d).

APAP overdose dramatically increased the hepatic mRNA expression of pro-inflammatory cytokine genes (*Il1b*, *Il6*, *Tnf*), accompanied by a limited increase in the anti-inflammatory cytokine gene (*Il10)*. Administration of Tec or PBHB moderately reversed these alterations, but the PBHB@Tec group exhibited a more pronounced reduction in pro-inflammatory cytokine expression and a notable increase in *Il1*0 mRNA levels (Fig. 2e). Consistent with mRNA expression patterns, hepatic protein levels of inflammatory cytokines interleukin-1β (IL-1β), interleukin-6 (IL-6), tumor necrosis factor-α (TNF-α), and interleukin-10 (IL-10) showed similar trends, with PBHB@Tec treatment demonstrating the most effective suppression of pro-inflammatory cytokines and elevation of IL-10 protein (Fig. 2f). Serum cytokine concentrations further corroborated these findings (Fig. 2g). Taken together, these findings demonstrate that PBHB@Tec treatment effectively attenuates the APAP-induced inflammatory response, as evidenced by the concurrent histological improvements and favorable modulation of cytokine profiles at the mRNA, protein, and serum levels.

**Fig. 3:**
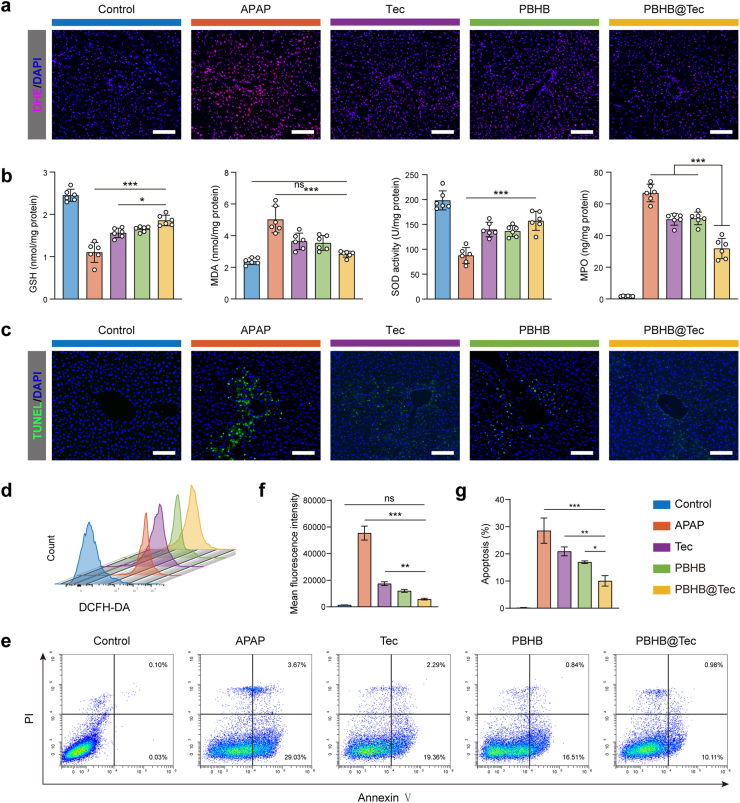
PBHB@Tec attenuates APAP-induced oxidative stress and hepatocyte apoptosis. (a) Representative DHE staining of liver tissues for ROS detection. Scale bar: 100 μm. (b) Quantification of hepatic antioxidant parameters, including GSH content, SOD activity, MDA and MPO levels (n = 6). (c) Representative TUNEL staining of liver tissues to assess apoptosis. Scale bar: 100 μm. (d) Flow cytometric analysis of ROS in primary hepatocytes using DCFH-DA. (e) Flow cytometric analysis of apoptosis in primary hepatocytes using Annexin V-FITC/PI staining. (f) Quantification of MFI of DCFH-DA (n = 3). (g) Quantification of the percentage of apoptotic cells (n = 3). Data are expressed as mean ± SD. ∗*p* < 0.05; ∗∗*p* < 0.01; ∗∗∗*p* < 0.001; ns, not significant.

APAP-induced hepatotoxicity is primarily driven by excessive ROS generation, which triggers oxidative stress. Dihydroethidium (DHE) staining demonstrated an appreciable increase in hepatic ROS levels following APAP exposure, whereas PBHB@Tec treatment notably reduced ROS accumulation (Fig. 3a and Fig. S6). Moreover, PBHB@Tec restored hepatic glutathione (GSH) content and superoxide dismutase (SOD) activity, and decreased malondialdehyde (MDA) and myeloperoxidase (MPO) levels, reflecting noteworthy mitigation of oxidative stress and inflammation (Fig. 3b). The restored redox balance consequently led to a substantial reduction in the number of terminal deoxynucleotidyl transferase-mediated dUTP nick end labeling (TUNEL)-positive cells in liver tissues from PBHB@Tec-treated mice (Fig. 3c and Fig. S6), underscoring its anti-apoptotic effect. To further validate the tissue-level observations at the cellular level, primary hepatocytes were isolated and subjected to flow cytometric analysis using intracellular ROS probe 2′,7′-dichlorodihydrofluorescein diacetate (DCFH-DA) and Annexin V-FITC/PI staining (Fig. 3d and e). The results were consistent with the hepatic tissue staining results (Fig. 3f and g), substantiating the robust antioxidative and cytoprotective efficacy of PBHB@Tec against APAP-induced oxidative stress and hepatocyte apoptosis.

### 2.3 PBHB@Tec alleviates ER stress and mitochondrial dysfunction *in vivo*

**Fig. 4:**
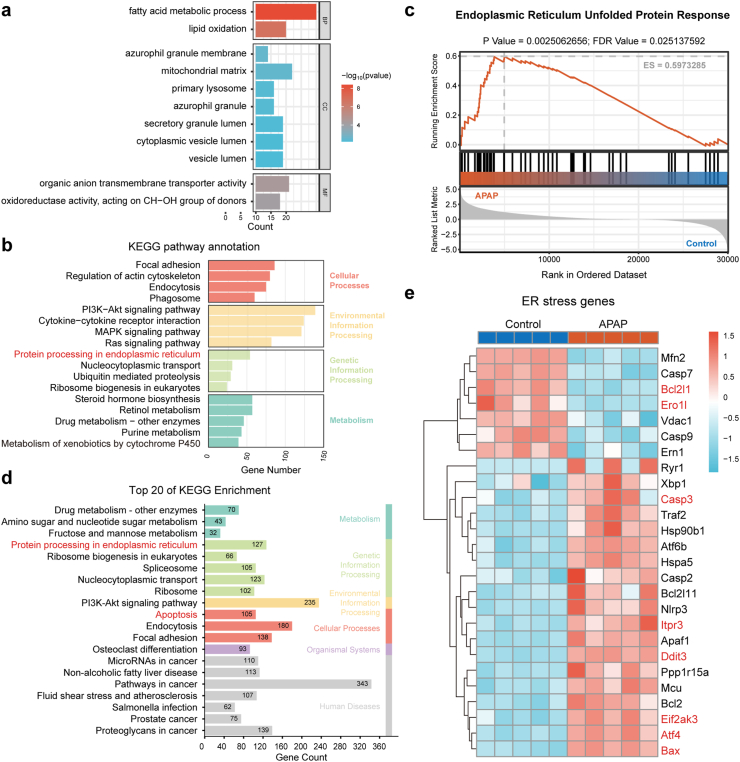
Transcriptomic analysis identifies ER stress activation as a key feature in APAP-induced liver injury. (a) GO enrichment analysis of liver transcriptomes from patients with APAP-induced acute liver failure and healthy controls (GSE74000 dataset). (b–e) Transcriptomic analyses of liver tissues from APAP-challenged mice and control mice, including: (b) KEGG pathway enrichment analysis of differentially expressed genes. (c) GSEA showing significant enrichment of the ER unfolded protein response pathway. (d) Top 20 KEGG-enriched pathways highlighting protein processing in ER and apoptosis-related pathways. (e) Heatmap of representative ER stress-related genes showing differential expression between control and APAP-treated mice.

To investigate the main physiological processes affected by AILI, a Gene Ontology (GO) enrichment analysis was performed on the transcriptomic data of liver samples from patients with APAP-induced ALF and healthy individuals from the GSE74000 dataset. The results indicated that damaged liver tissue exhibited disturbances in fatty acid and lipid metabolism, enhanced ER activity, and alterations in multiple processes related to oxidation-reduction (Fig. 4a). To further explore the relevance of these findings, the transcriptomic profiles of liver tissues from APAP-treated mice and healthy controls were analyzed. Kyoto Encyclopedia of Genes and Genomes (KEGG) pathway enrichment analysis highlighted “protein processing in endoplasmic reticulum” as a significantly enriched pathway (Fig. 4b), reinforcing the notion that ER stress and disrupted protein homeostasis are central components of the hepatic response to APAP overdose. Gene Set Enrichment Analysis (GSEA) revealed a statistically significant enrichment of the ER UPR pathway in APAP-challenged livers (False Discovery Rate = 0.025), further substantiating its role in the pathogenesis of AILI (Fig. 4c). Consistently, KEGG pathway enrichment rankings identified conspicuous involvement of protein processing in the ER and apoptosis pathways (Fig. 4d), suggesting that unresolved ER stress contributes to hepatocyte apoptosis. The heatmap of representative ER stress-related gene sets revealed that APAP exposure led to the activation of UPR and downstream mitochondria-mediated apoptotic cascades (Fig. 4e).

**Fig. 5:**
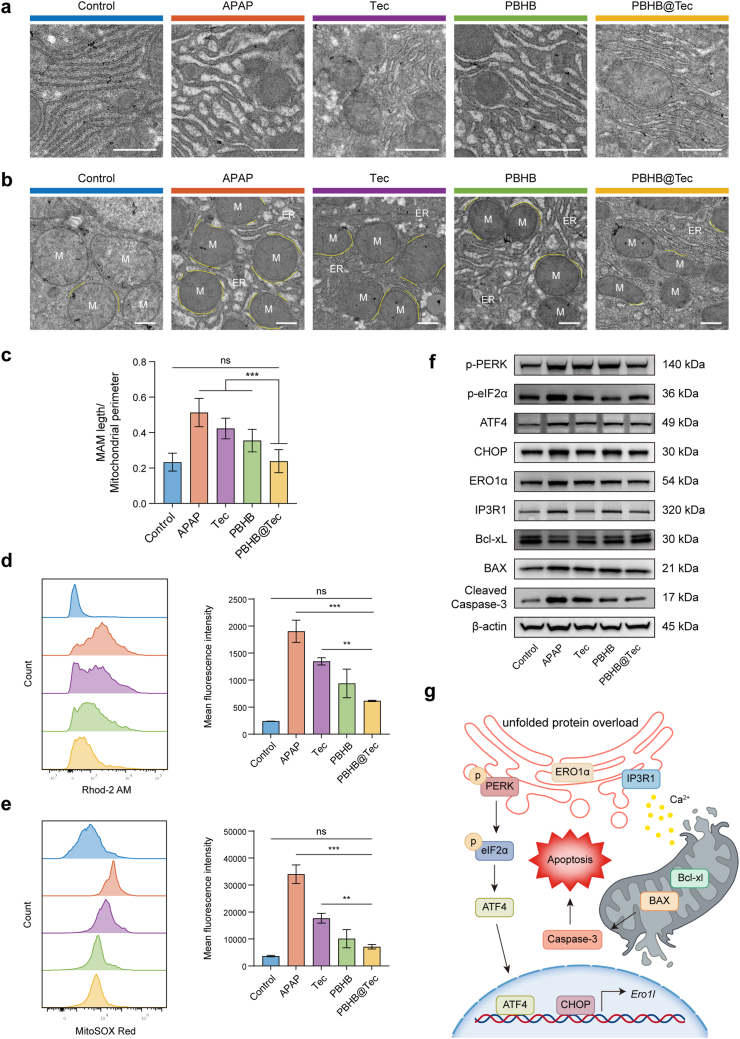
PBHB@Tec protects against ER stress and mitochondrial apoptosis in the APAP-induced acute liver injury mouse model. (a) Representative TEM images of liver sections at a magnification of 4300×, showing ultrastructural changes in ER morphology following different treatments. Scale bar: 1 μm. (b) Representative TEM images of liver sections at a magnification of 13500×, showing MAMs. Scale bar: 500 nm. ER (endoplasmic reticulum); M (mitochondria). The yellow line indicates the contact area between mitochondria and ER. (c) Quantification of MAM length normalized to mitochondrial perimeter. (d, e) Flow cytometry histograms and quantification of (d) mitochondrial calcium levels (Rhod-2 AM) and (e) mitochondrial superoxide levels (MitoSOX Red) in primary hepatocytes isolated from mouse liver tissues. (f) Western blot analysis of ER stress markers and mitochondrial apoptosis-related proteins. (g) Illustration of the molecular mechanism of ER stress-induced apoptosis regulated by the PERK–eIF2α–ATF4–CHOP signaling pathway and mitochondrial apoptosis regulators. Data are expressed as mean ± SD (n = 3). ∗*p* < 0.05; ∗∗*p* < 0.01; ∗∗∗*p* < 0.001; ns, not significant.

As a key organelle responsible for protein folding and calcium storage, the ER experiences increased protein folding demand during ER stress, which can lead to dysregulated calcium release and disruption of intracellular calcium homeostasis. To characterize these molecular events at the subcellular level, the ultrastructure of the ER and its physical interactions with mitochondria were examined using TEM. Following APAP intoxication, the ER of hepatocytes underwent significant morphological alterations, including dilation of the ER lumen, loosening of the ER membranes, disorganization of the ER network, and the formation of vesicle-like structures or vacuolization. Treatment with PBHB@Tec reduced ER lumen dilation, preserved membrane integrity, and improved organization of the ER network in hepatocytes (Fig. 5a). Beyond ER morphological changes, APAP exposure led to an increase in the abundance of mitochondria-associated ER membranes (MAMs) (Fig. 5b and c). MAMs are critical zones that facilitate direct calcium transfer from the ER to mitochondria, especially under stress conditions. Notably, PBHB@Tec treatment markedly reduced the abundance of MAMs, returning them close to baseline levels, indicating an effective limitation of pathological ER–mitochondria coupling. ER dilation and vesiculation compromise the function of calcium-binding chaperones, impairing ER calcium buffering capacity and triggering uncontrolled Ca^2+^ release into the cytosol. The increased cytosolic Ca^2+^ is efficiently transferred to mitochondria through MAMs via the VDAC-MCU axis [36,37]. Rather than the flux itself, it is the accumulation of Ca^2+^ within the mitochondrial matrix that triggers pathological consequences. Such accumulation activates tricarboxylic acid (TCA) cycle dehydrogenases, increasing the supply of reducing equivalents. This enhanced substrate availability accelerates electron transport chain (ETC) activity but simultaneously increases electron leakage, particularly at complexes I and III, thereby elevating mitochondrial ROS (mtROS) generation. Concurrently, excessive mitochondrial Ca^2+^ can trigger mPTP opening, leading to loss of membrane potential. This depolarization further exacerbates ETC dysfunction and amplifies mtROS production [38]. Additionally, mtROS oxidizes ER calcium-handling proteins, perpetuating ER calcium leakage, while also inactivating mitochondrial antioxidant defences, establishing a vicious feedforward cycle. Flow cytometric analysis of primary hepatocytes isolated from APAP-treated mice detected significantly elevated Rhod-2 AM and MitoSOX Red fluorescence signals (Fig. 5d and e), confirming mitochondrial Ca^2+^ overload and mtROS overproduction. The reduction in these two fluorescence signals upon PBHB@Tec treatment suggests that by limiting both mitochondrial calcium overload and excessive ROS production, PBHB@Tec helps restore calcium homeostasis and reduces the progression of cell injury under oxidative stress conditions.

The UPR triggered by ER stress functions cooperatively through three core pathways, among which the PERK-eIF2α-ATF4-CHOP axis acts as the central mediator of apoptosis and a key integrator of calcium–ROS crosstalk. Key genes encoding proteins of this axis and its downstream effectors exhibited significant differential expression following APAP challenge (Fig. 4e). These transcriptomic findings were further corroborated at the protein level by Western blot analysis. Decreased activation of the p-PERK–p-eIF2α–ATF4–CHOP axis and reduced expression of its downstream effectors, along with increased levels of the anti-apoptotic protein B-cell lymphoma-extra large (Bcl-xL) and reduced expression of the pro-apoptotic Bcl-2-associated X protein (BAX) were observed in PBHB@Tec-treated mice, collectively confirming that PBHB@Tec effectively attenuated APAP-induced ER–mitochondrial crosstalk and subsequent apoptosis (Fig. 5f and Fig. S7). To mechanistically contextualize these findings, the PERK-eIF2α-ATF4-CHOP signaling cascade and its contribution to ER–mitochondria crosstalk and cell fate were illustrated in Fig. 5g. Unfolded protein accumulation triggers PERK autophosphorylation, which phosphorylates eIF2α to attenuate global translation while selectively upregulating ATF4. ATF4-mediated CHOP upregulation transcriptionally activates ERO1α, which in turn promotes oxidative folding and oxidizes ER calcium-release channel IP3R1. Oxidation of IP3R1 enhances ER calcium leak, resulting in mitochondrial calcium overload and subsequent ROS generation. This feedforward loop is further amplified as mtROS oxidizes IP3R1, perpetuating calcium dysregulation. Concurrently, CHOP alters the Bcl-2 family balance by downregulating Bcl-xL and upregulating BAX, ultimately triggering mitochondrial outer membrane permeabilization and the activation of cysteine-aspartic protease-3 (Caspase-3)-dependent apoptosis. Collectively, this cascade establishes a mechanistic link between proteotoxic stress and cell death via tightly coupled ER-mitochondria signaling.

### 2.4 PBHB@Tec attenuates APAP-induced organelle dysfunction and apoptosis *in vitro*

**Fig. 6:**
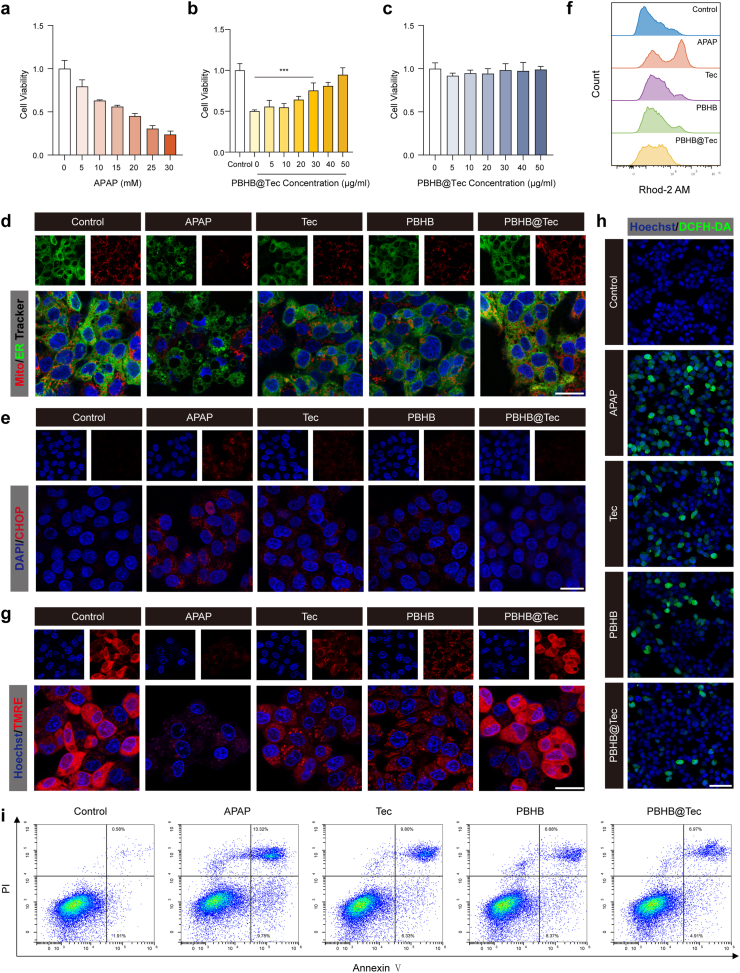
PBHB@Tec mitigates APAP-induced cytotoxicity and restores mitochondrial and ER homeostasis in HepG_2_ cells. (a) Cell viability of HepG_2_ cells treated with APAP (0–30 mM). (b) Cell viability of HepG_2_ cells treated with PBHB@Tec (0–50 μg/mL). (c) Cell viability of HepG_2_ cells co-treated with APAP (20 mM) and PBHB@Tec (0–50 μg/mL). (d) Representative confocal microscopy images showing mitochondrial (MitoTracker, red) and ER (ER Tracker, green) morphology in HepG_2_ cells. Scale bar: 20 μm. (e) Immunofluorescence staining for CHOP (red) with DAPI nuclear counterstaining (blue) in HepG_2_ cells. Scale bar: 20 μm. (f) Flow cytometry histograms showing mitochondrial calcium levels in HepG_2_ cells stained with Rhod-2 AM. (g) MMP assessment by TMRE staining in HepG_2_ cells. Scale bar: 20 μm. (h) Fluorescent imaging of ROS levels detected by DCFH-DA (green) and nuclei counterstained with Hoechst (blue) in HepG_2_ cells. Scale bar: 50 μm. (i) Flow cytometry analysis of apoptosis by Annexin V-FITC/PI staining in HepG_2_ cells. Data are expressed as mean ± SD (n = 3). ∗*p* < 0.05; ∗∗*p* < 0.01; ∗∗∗*p* < 0.001; ns, not significant.

To establish an *in vitro* model of APAP-induced hepatocellular injury, human hepatocellular carcinoma cells (HepG_2_) cells were treated with increasing concentrations of APAP (0–30 mM) for 24 h to determine the optimal dose (Fig. 6a). Based on the dose-response results, 20 mM APAP was selected as it induced significant cellular injury while preserving sufficient viability for subsequent therapeutic evaluation. HepG_2_ cells were then treated with various concentrations of PBHB@Tec in the presence of 20 mM APAP. PBHB@Tec treatment enhanced cell viability in a dose-dependent manner, with 30 μg/mL identified as the minimal effective concentration for further studies (Fig. 6b). PBHB@Tec did not exhibit cytotoxicity within the tested concentration range (Fig. 6c), indicating favorable biocompatibility.

Building on the *in vivo* findings, mitochondria and ER morphology in HepG_2_ cells were assessed by confocal microscopy following co-staining with Mito-Tracker and ER-Tracker (Fig. 6d). APAP treatment caused notable disruption to both organelles, characterized by fragmented and punctate mitochondria and a distorted, condensed ER network, in contrast to the elongated and interconnected mitochondrial network and reticular ER structure surrounding the nuclei seen in untreated cells. Administration of PBHB@Tec substantially alleviated APAP-induced organelle damage, as demonstrated by partial preservation of mitochondrial network integrity and restoration of a more reticular ER morphology. Immunofluorescence staining for CHOP, a key transcription factor mediating ER stress, was performed to further evaluate ER stress activation in HepG_2_ cells (Fig. 6e). CHOP expression was strongly upregulated following APAP exposure, whereas treatment with PBHB@Tec markedly reversed this upregulation, suggesting effective attenuation of ER stress. Given that mitochondrial calcium overload is a downstream consequence of excessive ER stress, mitochondrial calcium levels were evaluated using the Rhod-2 AM probe (Fig. 6f and Fig. S8). Under APAP challenge, Rhod-2 AM fluorescence intensity increased substantially. Cells treated with PBHB@Tec maintained much lower fluorescence signals, which indicating restored mitochondrial calcium homeostasis. This highlights the effectiveness of PBHB@Tec in stabilizing mitochondrial function by preventing calcium dysregulation during APAP-induced stress. The disruption of mitochondrial membrane potential (MMP) is an early indicator of mitochondrial dysfunction and often leads to excessive mtROS production. Elevated ROS levels can further damage cellular components, amplifying oxidative stress and triggering apoptotic signaling pathways. Tetramethylrhodamine ethyl ester (TMRE) staining revealed a significant loss of MMP in APAP-treated cells, which was accompanied by increased ROS generation as indicated by DCFH-DA staining. Conversely, treatment with PBHB@Tec effectively restored mitochondrial membrane polarization and reduced ROS accumulation (Fig. 6g and h). Flow cytometric analysis revealed that APAP markedly elevated the proportion of apoptotic cells, reflecting the culmination of ER stress and mitochondrial dysfunction. This increase was significantly mitigated by PBHB@Tec treatment (Fig. 6i and Fig. S9). Thus, by maintaining organelle integrity and cellular redox balance, PBHB@Tec effectively inhibits the execution of apoptosis, highlighting its potential as a therapeutic strategy against AILI.

### 2.5 PBHB@Tec exhibits enhanced hepatocyte targeting and reduced macrophage clearance

**Fig. 7:**
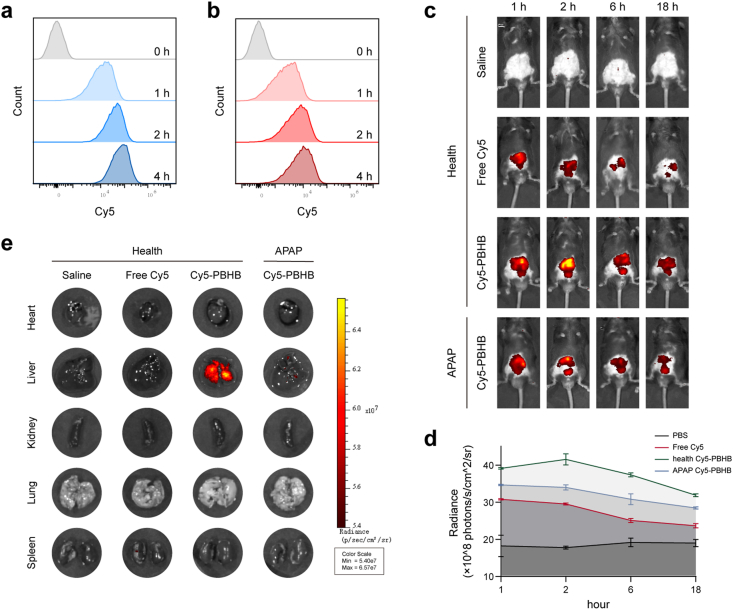
Cellular uptake and biodistribution of PBHB *in vivo*. (a, b) Flow cytometry analysis of cellular uptake of Cy5-PBHB by HepG_2_ cells and RAW264.7 cells after incubation for 0, 1, 2, and 4 h. (c) Representative *in vivo* fluorescence images showing the biodistribution of free Cy5 and Cy5-PBHB in healthy and APAP-induced acute liver injury model mice; saline-injected mice served as controls. (d) Quantification of ROI fluorescence intensity. (e) Representative ex vivo fluorescence images of major organs (heart, liver, spleen, lungs, kidneys) harvested at 18 h post-injection. Data are expressed as mean ± SD (n = 3).

To investigate the cellular uptake dynamics of PBHB, HepG_2_ hepatocytes and RAW 264.7 macrophages were incubated with Cy5-labeled PBHB for 0, 1, 2, and 4 h, followed by flow cytometry to quantitatively assess fluorescence intensity as a measure of nanoparticle internalization (Fig. 7a and b). Both cell types exhibited a time-dependent increase in Cy5 fluorescence. However, HepG_2_ cells showed a more prominent and sustained accumulation of PBHB over 4 h compared to RAW 264.7, which displayed relatively lower fluorescence intensity (Fig. S10). The differential uptake patterns indicated that PBHB preferentially targets hepatocytes, the principal parenchymal liver cells, while effectively minimizing clearance by macrophages—major components of the liver's reticuloendothelial system responsible for nanoparticle sequestration and clearance. Such selective cellular targeting is critical for enhancing therapeutic payload delivery directly to injured hepatocytes. These findings underscore the design advantage of PBHB in evading rapid clearance by Kupffer cells and increasing hepatic accumulation. This selective interaction behavior lays a mechanistic foundation for the observed therapeutic benefits in AILI mouse model.

To evaluate the *in vivo* biodistribution and hepatic targeting efficiency of PBHB under physiological conditions, three groups of healthy mice were intravenously injected via the tail vein with saline, free Cy5 dye, and Cy5-labeled PBHB, respectively. Additionally, AILI model mice were injected with Cy5-labeled PBHB. *In vivo* fluorescence imaging was performed at 1, 2, 6, and 18 h post-injection to monitor the biodistribution and retention of nanoparticles (Fig. 7c). The fluorescence quantification results showed that PBHB efficiently targets and accumulates in the liver, exhibiting prolonged residence time compared to free Cy5 dye (Fig. 7d). Major organs were harvested at 18 h to measure residual fluorescence (Fig. 7e). Persistent fluorescence signals were visible in the healthy liver after PBHB administration, whereas no obvious fluorescence was observed in the APAP-treated liver. This difference aligns with the lower hepatic fluorescence observed in AILI mice compared with healthy controls throughout the imaging period. This reduction may be partly due to elevated ROS levels in the injured liver microenvironment, which accelerate nanoparticle consumption via their ROS-scavenging activity. However, other physiological changes associated with severe hepatic injury—such as reduced hepatic blood flow or damage to the sinusoidal endothelial architecture—could also decrease hepatic nanoparticle accumulation efficiency. These factors may collectively account for the observed reduction in fluorescence intensity in the AILI group. Fluorescence signals in other major organs were minimal, demonstrating favorable liver-specific targeting and limited off-target distribution. No adverse effects, such as pulmonary embolism or overt toxicity, were observed during the administration period. The hemolysis assay confirmed excellent hemocompatibility of PBHB (Fig. S11). Together with the demonstrated 24 h physiological stability (Fig. S12), which aligns with the window of hepatic accumulation, these results indicate a favorable safety profile. No histopathological abnormalities were observed in the heart, lung, spleen, or kidney tissues harvested at 24 h post APAP injection as assessed by HE staining (Fig. S13), further confirming the good biocompatibility and biosafety of the nanoparticles.

## 3 Conclusions

In summary, we present a ROS-responsive nanoplatform (PBHB@Tec) for site-specific delivery and antioxidant intervention against APAP-induced hepatotoxicity. The nanoparticles directly scavenge ROS and selectively release Tec within the oxidative hepatic microenvironment. Comprehensive studies demonstrated that PBHB@Tec alleviates ER stress, preserves mitochondrial function, and attenuates liver injury by disrupting the feedforward ER–mitochondrial Calcium/ROS signaling cascade. Biodistribution confirmed preferential hepatic accumulation with no off-target toxicity.

By progressing from *in vivo* therapeutic discovery to mechanistic elucidation at the cellular level, we identified biologically relevant pathways within the pathophysiological hepatic microenvironment and thereby enhanced the mechanistic depth and translational relevance of the findings. This study provides a generalizable strategy for transforming natural molecules with limited bioavailability into viable therapies for liver diseases and exemplifies a new paradigm for precision nanotherapeutics in hepatoprotection. However, the precise sites of action for individual components remain to be fully elucidated. Future studies employing component-specific molecular interventions or genetic models will be necessary to delineate the specific pathway nodes targeted by each component.

## 4 Materials and methods

### 4.1 Synthesis of PBHB

The PBHB was synthesized by a facile three-step method. Briefly, 10 g pullulan and 6 g succinic anhydride were dissolved in milli-Q and heated to 70 °C. The pH of the mixture was adjusted within 7.5–8.0 via NaOH (2 M). After 12 h reaction, the product was precipitated and washed by ethanol for 3 times and collected via 8000 rpm centrifugation for 10 min. The intermediate PSu was obtained through lyophilization. Then, 3 g PSu, 270 μL pyridine and 500 μL n-butyric anhydride were dissolved in 10 mL formamide. The mixture was heated to 60 °C and reacted for 15 h into milli-Q water and dried in vacuum oven at room temperature. Finally, HBPAK was conjugated to PB in DMSO via EDC/NHS at room temperature for 1 h and the product PBHB was precipitated with methyl tert-butyl ether and vacuum-dried. The molecular structure of PBHB and its intermediates were characterized by ^1^H NMR (AVANCE NEO 400, Bruke).

### 4.2 Preparation of nanoparticles

Nanoparticle size and distribution were optimized by systematically varying PVA concentration (0.5 %, 1 %, and 2 %) and PBHB content in the organic phase. The 100 mg PBHB and 10 mg Tec were dissolved in 11 mL DMSO. The solution was injected into 1 % PVA solution (water phase) in drop-wise with syringe pump. The nanoparticles were collected by centrifugation at 10000 rpm for 10 min and washed with milli-Q for 3 times. The precipitation was lyophilized to obtain the PBHB@Tec. The PB nanoparticles were fabricated via the same process without Tec.

The particle size and its distribution as well as zeta potential were characterized by dynamic laser scatter (DLS, Zetasizer, Malvern). Nanoparticle morphology was examined by TEM (HT7700, Hitachi), and the elemental composition was determined by XPS (Axis Supra+, Kratos).

### 4.3 Drug release profile of nanoparticles

The loading efficiency (LE) was calculated using Equation (1). Tec-loaded nanomedicines were dispersed in PBS containing 0.1 % Tween 80 and incubated at 37 °C, 120 rpm; cumulative release was quantified at predetermined intervals. The PBHB@Tec + H_2_O_2_ group was exposed to buffer supplemented with 10 mM H_2_O_2_, whereas the PBHB group remained H_2_O_2_-free. Released Tec concentrations were determined by UV–vis spectrophotometry (UV-2600i, Shimadzu).

(1) LE = Amount of drug loaded/Total amount of nanoparticles

### 4.4 Antioxidant property of the nanoparticles

The nanoparticles were re-dispersed in milli-Q water and serially diluted to 1 mg/mL, 500 μg/mL, 250 μg/mL, 125 μg/mL and 62.5 μg/mL. Total antioxidant activity and H_2_O_2_-scavenging capacity were quantified at 37 °C for 30 min using an ABTS kit and KI/H_2_O_2_ (1 M/100 μM), respectively. To monitor ROS-responsive degradation, the nanoparticles were incubated with 10 mM H_2_O_2_, and aliquots withdrawn at defined time points were imaged by TEM to visualize morphological evolution.

### 4.5 Cells and animals

HepG_2_ and RAW264.7 macrophage cells were obtained from Pricella Biotechnology Co., Ltd. (Wuhan, China). Cells were cultured in high-glucose Dulbecco's Modified Eagle's Medium (DMEM) supplemented with 10 % fetal bovine serum and 1 % penicillin-streptomycin, incubated at 37 °C in a humidified atmosphere containing 5 % CO_2_. Male C57BL/6 mice aged 6–8 weeks were used for all *in vivo* experiments. Animals were housed under standard laboratory conditions with free access to food and water.

### 4.6 *In vivo* evaluation of therapeutic effects

Mice were housed under specific pathogen-free (SPF) conditions for 7 days to acclimate and then randomly assigned into five groups (n = 6 per group): the Control group, the APAP group, the Tec group, the PBHB group, and the PBHB@Tec group. ALI was induced by intraperitoneal injection of 300 mg/kg APAP in all groups except control, which received an equivalent volume of saline. At 6 h after APAP injection, mice in the Tec, PBHB@Tec and PBHB groups were intravenously injected with 200 μL of Tec (25 mg/kg), PBHB@Tec (Tec-equivalent dose of 25 mg/kg) or PBHB (at a nanoparticle dose equivalent to that in the PBHB@Tec group), respectively. The control and APAP groups received equal volumes of saline. After 24 h, blood, liver, and other major organs were harvested for further analysis.

### 4.7 Statistical analysis

All statistical data are expressed as the mean ± standard deviation (SD) and were analyzed using GraphPad Prism software (version 9.0.0). Differences between two groups were evaluated using unpaired Student's t-test, while comparisons among three or more groups were analyzed using one-way analysis of variance (ANOVA) followed by Tukey's post hoc test for pairwise comparisons. All data were collected from a minimum of three independent experiments. Differences were considered statistically significant for ∗*p* < 0.05, ∗∗*p* < 0.01, and ∗∗∗*p* < 0.001, while ns represented not significant.

Comprehensive details of additional materials and methodological procedures are available in the Supporting Information.

## CRediT authorship contribution statement

**Yaqi Zhang:** Writing – original draft, Validation, Methodology, Formal analysis, Conceptualization. **Zeyuan Jin:** Writing – original draft, Validation, Methodology, Formal analysis, Conceptualization. **Lvwan Xu:** Investigation, Data curation. **Zilong Zhong:** Investigation, Data curation. **Xinyu Wang:** Investigation, Data curation. **Changyou Gao:** Writing – review & editing, Supervision, Funding acquisition. **Lanjuan Li:** Writing – review & editing, Supervision, Funding acquisition.

## Ethics approval and consent to participate

All animal procedures were approved by the Institutional Animal Care and Use Committee (IACUC) of The First Affiliated Hospital, Zhejiang University School of Medicine (approval number: 2025026) and performed in accordance with the Guide for the Care and Use of Laboratory Animals. Experimental protocols were designed to minimize animal suffering and to adhere to the principles of the 3Rs (Replacement, Reduction, and Refinement).

## Declaration of competing interest

The authors declare that they have no known competing financial interests or personal relationships that could have appeared to influence the work reported in this paper.
