# Supplementary material for: ROS-scavenging nanoparticles loaded with tectorigenin protect against acetaminophen-induced hepatotoxicity by interrupting the calcium/ROS-mediated pathogenic endoplasmic reticulum–Mitochondrial signaling cascade
Source: Bioact Mater. 2025 Dec 13;58:408–21. doi: 10.1016/j.bioactmat.2025.12.016 (PMC12765072; doi:10.1016/j.bioactmat.2025.12.016)
Supplement: Multimedia component 1 [file mmc1.docx]

**Supplementary Information**

**Supplementary Methods**

1. ***In vitro* experiments**

**1.1 *In Vitro* Tec Release Profile from PBHB@Tec by Dialysis**

Briefly, 10 mg of PBHB@Tec nanoparticles were dispersed in 5 mL of PBS containing 0.02% PS80 and loaded into a dialysis bag (molecular weight cutoff: 500 Da). The bag was then placed in 20 mL of the same buffer solution and incubated at 37 °C under gentle shaking at 120 rpm. At predetermined time intervals, 1 mL of the external buffer was sampled and replaced with an equal volume of fresh buffer. The concentration of released Tec in the sampled solution was quantified by UV-vis spectroscopy (UV-2600i, Shimadzu). The cumulative release percentage of Tec at each time point was calculated as the ratio of the amount released to the initial drug loading in the PBHB@Tec nanoparticles.

**1.2 Cell Viability Assessment**

To identify the APAP concentrations that induce hepatocyte injury in cell models, HepG_2_ cells were seeded into 96-well plates at a density of 5 × 10³ cells per well and cultured for 24 h, then treated with a concentration gradient of APAP (5, 10, 15, 20, 25, and 30 mM) for 24 h. Cells at the same density were incubated with different concentrations of PBHB@Tec (5, 10, 20, 30, 40, and 50 μg/mL) for 24 h to assess the cytotoxicity of the nanoparticles. Cell viability was evaluated using the CCK-8 assay. To determine the therapeutic efficacy of PBHB@Tec, HepG_2_ cells were seeded into 96-well plates (5 × 10³ cells/well) for 24 h and treated with 20 mM APAP for 6 h to induce hepatocyte injury, followed by addition of PBHB@Tec (5, 10, 20, 30, 40, and 50 µg/mL). The total duration of treatment was 24 h, at the end of which cell viability was measured via CCK-8 assay according to the manufacturer’s instructions.

**1.3 Immunofluorescence staining**

HepG_2_ cells (1 × 10⁵ cells/well) were seeded onto coverslips in 24-well plates and cultured for 24 h. Following 6 h treatment with 20 mM APAP, Tec, PBHB, or PBHB@Tec was added to the medium for an additional 18 h incubation. After PBS washing, cells were fixed with 4% paraformaldehyde and permeabilized with 0.1% Triton X-100. Primary antibody incubation (anti-CHOP, 15204-1-AP, Proteintech) was performed overnight at 4 °C, then cells were labeled with Alexa Fluor 647-conjugated secondary antibody (A-31573, Invitrogen) for 1 h at room temperature and counterstained with DAPI. Finally, coverslips were transferred to glass slides, mounted with anti-fade medium, and imaged by confocal laser scanning microscope (FV3000, Olympus).

**1.4 Flow cytometry**

Cell apoptosis was determined using an Annexin V-FITC/PI Apoptosis Detection Kit (E-CK-A211, Elabscience) and intracellular calcium levels were detected using Rhod-2 AM (40776ES72, Yeasen). Briefly, HepG_2_ cells were seeded in 12-well plates at 2.5 × 10^4^ cells/well for 24 h, then pretreated with 20 mM APAP for 6 h before adding Tec, PBHB, or PBHB@Tec for 18 h. After incubation, cells were harvested using trypsin (without EDTA) and washed twice.

For apoptosis assay, the harvested cells were resuspended in 100 µL binding buffer, then stained with 5 µL Annexin V-FITC and 5 µL PI for 15 min at room temperature in the dark. Cells were analyzed within 1 h by flow cytometry (CytoFLEX, Beckman Coulter).

For intracellular calcium measurement, cells were loaded with 5 μM Rhod-2 AM in HBSS buffer at 37 °C for 30 min, washed twice, and incubated for an additional 20 min to allow complete dye de-esterification. Cells were analyzed immediately by flow cytometry (LSRFortessa X20, BD Biosciences).

**1.5 Intracellular ROS detection**

Intracellular ROS levels were measured using the fluorescent probe DCFH-DA (S0033, Beyotime). HepG_2_ cells were seeded in glass-bottom 6-well plates (5×10⁴ cells/well) and cultured for 24 h. After 6 h of treatment with 20 mM APAP, Tec, PBHB, or PBHB@Tec was added to the medium for another 18 h of incubation. A staining solution containing 10 μM DCFH-DA and 5 μg/mL Hoechst 33342 was added to each well and incubated for 20 min at 37 °C in the dark. Confocal imaging was performed using a Leica STELLARIS 5 microscope with a 40× objective.

**1.6 Mitochondrial and Endoplasmic Reticulum Staining**

HepG_2_ cells were seeded in 15 mm glass-bottom confocal dishes at a density of 1.5×10⁵ cells/dish and cultured for 24 h. After 6 h of treatment with 20 mM APAP, the cells were further treated with Tec, PBHB, or PBHB@Tec in the medium for another 18 h. After treatment, cells were stained with a dye mixture containing 1 μM ER-Tracker Green (C1042, Beyotime) and 200 nM Mito-Tracker Red CMXRos (C1049B, Beyotime) at 37 °C for 30 min, followed by nuclear staining with Hoechst 33342 for 10 min. The morphological changes of ER and mitochondria were observed using a Leica STELLARIS 5 confocal microscope.

**1.7 Mitochondrial Membrane Potential Assessment**

HepG_2_ cells were seeded in 15 mm glass-bottom confocal dishes at a density of 1×10⁵ cells/dish and cultured for 24 h. After 6 h of treatment with 20 mM APAP, the cells were further treated with Tec, PBHB, or PBHB@Tec in the medium for another 18 h. After treatment, cells were stained with 5 μM TMRE at 37 °C for 30 min, followed by nuclear staining with Hoechst 33342 for 10 min. The mitochondrial membrane potential (MMP) was visualized using a Leica STELLARIS 5 confocal microscope

**1.8 Cellular Uptake of Cy5-Labeled PBHB**

HepG_2_ and RAW264.7 cells were seeded in 12-well plates at 2.5 × 10⁴ cells/well and cultured for 24 h. Cells were incubated with 50 μg/mL Cy5-labeled PBHB for 0, 1, 2, or 4 h at 37 °C. After washing three times with PBS to remove unbound nanoparticles, cells were harvested and analyzed by flow cytometry (CytoFLEX, Beckman Coulter). PBHB uptake was quantified by MFI of Cy5.

**1.9 Hemolysis Assay**

Whole blood was collected from healthy mice using K2-EDTA anticoagulation tubes and gently mixed by inversion. The blood samples were centrifuged at 1500 rpm for 10 min at 4 °C, and the plasma was carefully removed. The remaining red blood cells (RBCs) were washed by saline until the supernatant became colorless. 20 µL of RBCs was gently mixed by pipetting with 1mL of test solution in a 1.5 mL Eppendorf tube. The experimental groups consisted of PBHB@Tec dispersed in saline at various concentrations (10, 25, 50, and 100 μg/mL). Saline served as the negative control, and deionized water as the positive control. All samples were incubated at 37 °C for 3 h. After incubation, the samples were centrifuged at 2500 rpm for 10 min at 4 °C. The supernatant from each sample was transferred to a 96-well plate and the absorbance at 540 nm was measured using a microplate reader (SpectraMax ABS, Molecular Devices).

**2. *In vivo* experiments**

**2.1 Determination of Serum Biochemical Indicators**

Serum alanine aminotransferase (ALT) and aspartate aminotransferase (AST) were measured using an automated biochemical analyzer (Hitachi 3100, Japan).

**2.2 Histopathological Analysis**

Heart, liver, spleen, lung, and kidney samples from mice were fixed in 4% paraformaldehyde, embedded in paraffin, sectioned into 3–5 μm-thick slices, and subsequently stained with hematoxylin and eosin (H&E) for histopathological examination.

To assess hepatocyte apoptosis, liver sections were subjected to TUNEL staining using a commercial kit (A111, Vazyme), following the manufacturer’s protocol.

Freshly harvested liver tissues were embedded in optimal cutting temperature (OCT) compound and frozen at -80 °C. Cryosections (8–10 µm) were prepared and stained with dihydroethidium (DHE) to evaluate ROS levels.

All stained sections were scanned using an automatic digital slide scanner (Pannoramic 250 FLASH III, 3DHISTECH) and analyzed using CaseViewer software (version 2.4.0, 3DHISTECH).

**2.3 Serum Cytokine Profiling**

Serum levels of IL-1β, IL-6, IL-10, and TNF-α were quantified using commercial ELISA kits (IL-1β: DKW12-2012; IL-6: DKW12-2060; IL-10: DKW12-2100; TNF-α: DKW12-2710, all from Dakewe). Briefly, 96-well plates pre-coated with specific capture antibodies were utilized, and the assays were performed according to the manufacturer’s instructions. The optical density (OD) was measured at 450 nm using a microplate reader (SpectraMax ABS, Molecular Devices), and cytokine concentrations were calculated based on standard curves.

**2.4 Measurement of Hepatic Cytokine Protein Levels**

Liver tissues were homogenized in PBS (10%, w/v) and centrifuged at 12 000 × g for 10 min at 4 °C. The supernatants were collected for analysis. Hepatic levels of IL-1β, IL-6, IL-10, and TNF-α were measured using ELISA kits (IL-1β: DKW12-2012; IL-6: DKW12-2060; IL-10: DKW12-2100; TNF-α: DKW12-2710; all from Dakewe). Briefly, 96-well plates pre-coated with specific capture antibodies were utilized, and the assays were performed according to the manufacturer’s instructions. The optical density (OD) was measured at 450 nm using a microplate reader (SpectraMax ABS, Molecular Devices), and cytokine concentrations were calculated based on standard curves. Results were normalized to total protein content determined by the BCA protein assay (BL521A, Biosharp) and expressed as pg/mg protein.

**2.5 Determination of Oxidative Stress Indicators**

Liver tissues were homogenized in PBS (10%, w/v) and centrifuged at 12,000 × g for 10 min at 4 °C. The supernatants were used to measure oxidative stress markers, including MDA, GSH, SOD (A003-1-2, A006-2-1, A001-3-2; Nanjing Jianchen) and MPO (EK0943; Boster), using commercial assay kits. Results were normalized to total protein content determined by the BCA protein assay (BL521A, Biosharp).

**2.6 RNA extraction and quantitative real-time PCR (qRT–PCR)**

Total RNA from mouse liver was extracted using TRIzol reagent (Invitrogen), and cDNA was synthesized with a reverse transcription kit (RR036, Takara). The forward and reverse primer sequences for the target genes are provided in Table S1. qRT-PCR was performed using TB Green Premix Ex Taq (RR820, Takara) on a 7500 Real-Time PCR system (Applied Biosystems). Relative expression levels were normalized to *Actb* and calculated using the 2^⁻ΔΔCt^ method.

**2.7 Analysis of open-access Datasets and RNA Transcriptome Sequencing (RNA-seq)**

The transcriptomic data of liver samples from patients with APAP-induced acute liver failure and healthy individuals was retrieved from the Gene Expression Omnibus (GEO) database (dataset GSE74000) and further subjected to enrichment analysis. For RNA-seq, total RNA was extracted from mouse liver tissues of the control and APAP-treated groups. Paired-end sequencing (2 × 150 bp, PE150) was performed on an Illumina NovaSeq™ 6000 platform (LC-Bio Technology Co., Ltd., Hangzhou, China) according to the manufacturer’s instructions.

**2.8 Transmission Electron Microscopy (TEM) Analysis of Hepatic Ultrastructure**

Liver tissues were trimmed into 1-mm³ blocks and fixed in 2.5% glutaraldehyde at 4 °C overnight. Subsequently, the tissues were post-fixed in 1% osmium tetroxide and stained with 2% aqueous uranyl acetate. After dehydration through a graded ethanol series, the samples were embedded in epoxy resin. Ultra-thin sections were prepared using an ultramicrotome (EM UC7, Leica Microsystem) and examined using a transmission electron microscope (Talos L120C, Thermo Scientific).

**2.9 Flow cytometry**

Fresh mouse liver tissue was minced and digested with tissue dissociation solution (abs9482, Absin) at 37 °C for 30 min with 180-rpm agitation. The cell suspension was filtered through a 70-µm strainer and purified through 35% Percoll gradient centrifugation to enrich for hepatocytes by removing non-parenchymal cells and lipids. After red blood cell lysis and PBS washing, hepatocytes were resuspended in DPBS for staining.

For the apoptosis assay, hepatocytes were stained using an Annexin V-FITC/PI Apoptosis Detection Kit (E-CK-A211, Elabscience) following the manufacturer’s instructions and analyzed within 1 h by flow cytometry (CytoFLEX, Beckman Coulter).

For intracellular calcium measurement, hepatocytes were loaded with 5 μM Rhod-2 AM (40776ES72, Yeasen) in HBSS buffer at 37 °C for 30 min, washed twice, and incubated for an additional 20 min to allow complete dye de-esterification. Cells were analyzed immediately by flow cytometry (LSRFortessa X20, BD Biosciences).

For detection of cellular ROS and mitochondrial superoxide, hepatocytes were incubated with a DCFH-DA (S0033, Beyotime) and MitoSOX Red (S0061, Beyotime) mixture at 37 °C for 30 min and analyzed by flow cytometry (CytoFLEX, Beckman Coulter).

**2.10 Western blotting analysis**

Total protein was extracted from homogenized liver tissues using RIPA lysis buffer containing protease and phosphatase inhibitors. Protein concentration was determined using a BCA protein assay kit (BL521A, Biosharp). Equal amounts of protein were resolved by SDS-PAGE and transferred to PVDF membranes. After blocking with QuickBlock buffer (P0252, Beyotime), the membranes were incubated with primary antibodies at 4 °C overnight and HRP-conjugated secondary antibodies at room temperature for 60 minutes. Signals were detected using ECL reagent (BL520A, Biosharp) and imaged with a chemiluminescence imaging system (JP-K300plus, JIAPENG). Antibody catalog numbers are listed in Table S2.

**2.11 *In vivo* biodistribution study**

Healthy mice were given tail vein injections of 200 µL saline, 1 mg/mL free Cy5.5, or 1 mg/mL Cy5.5-PBHB. Mice that received an intraperitoneal injection of 300 mg/kg APAP 6 h previously were then injected with an equal volume of 1 mg/mL Cy5.5-PBHB via the tail vein. At 1, 2, 6, and 18 h post-injection, the biodistribution of fluorescence was measured using an *in vivo* imaging system (IVIS Spectrum, PerkinElmer). Representative mice were sacrificed for collection of the heart, liver, lungs, kidneys, and spleen, and fluorescence from these organs was also measured.

**Supplementary Figures**

**
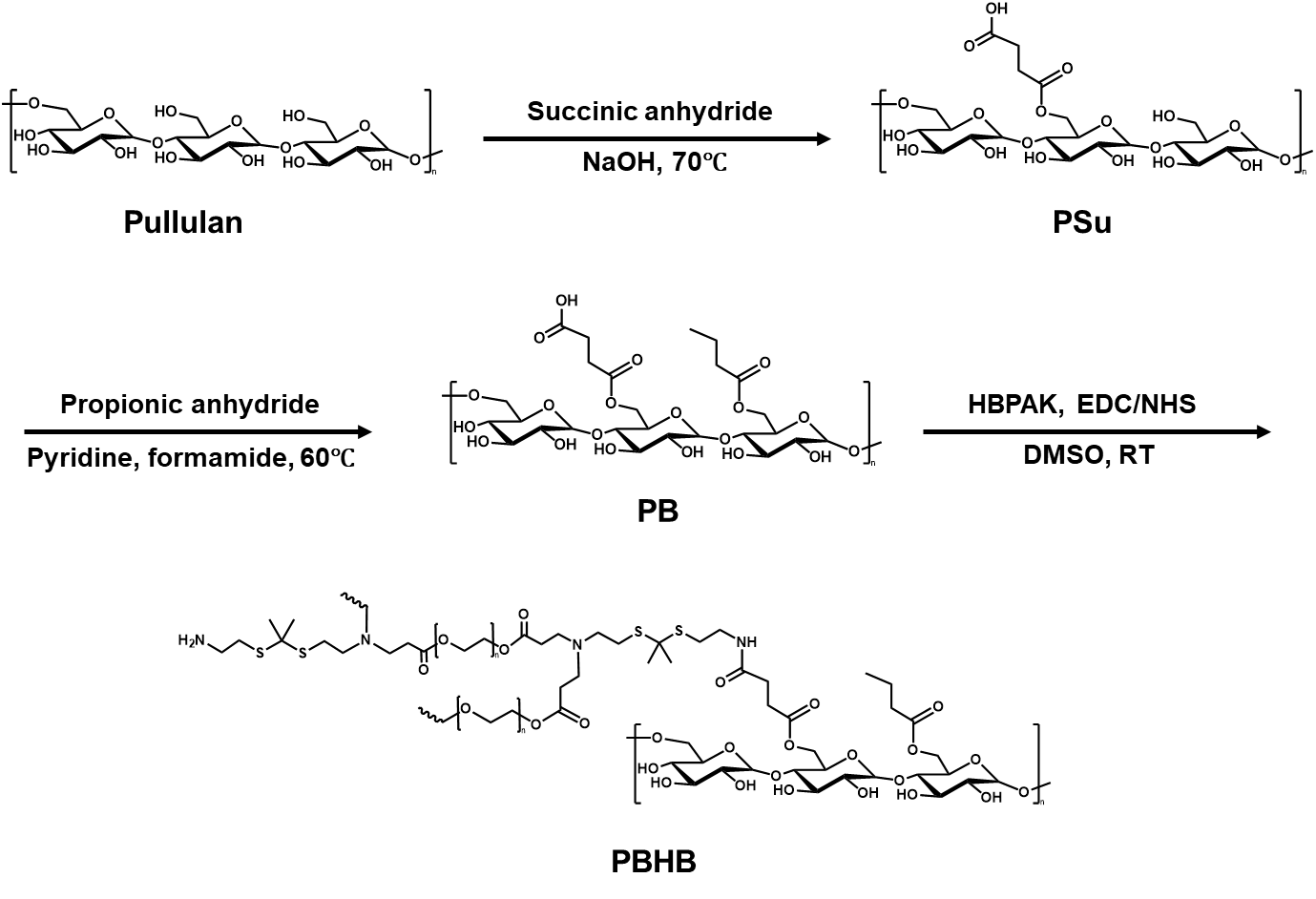
**

**Scheme 1.** Synthetic route of antioxidant polysaccharide PBHB.

**
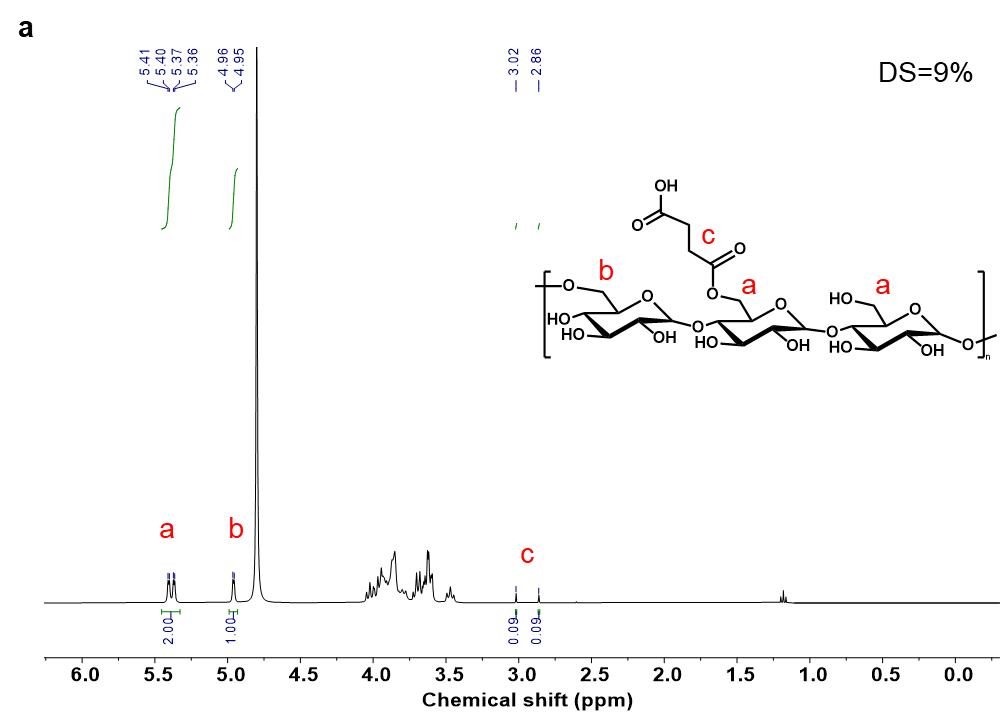
**

**
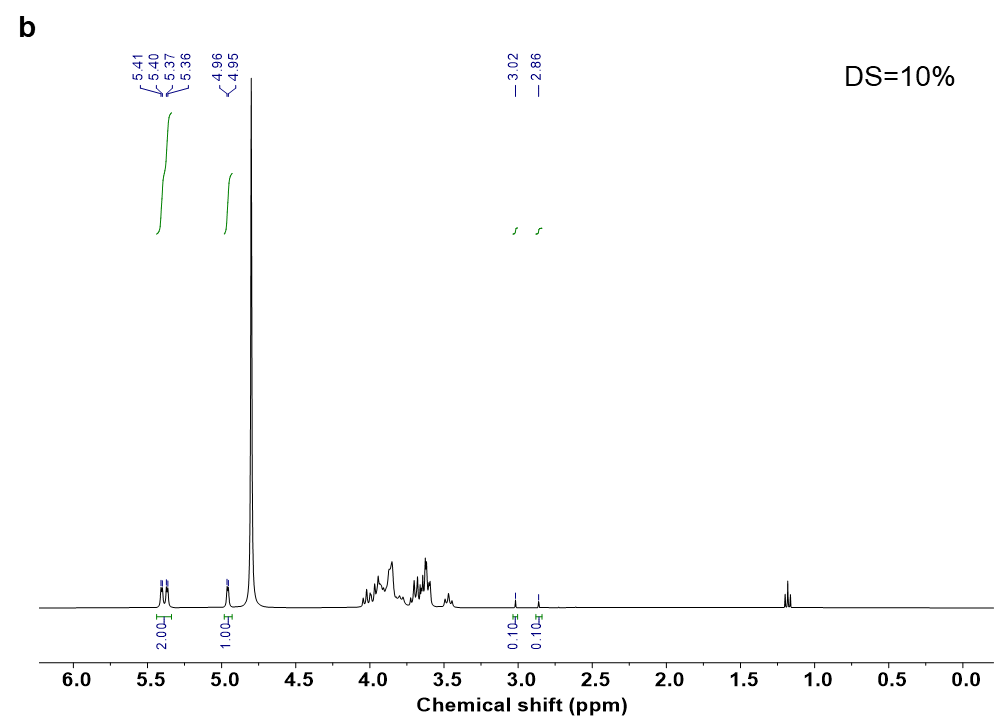
**

**
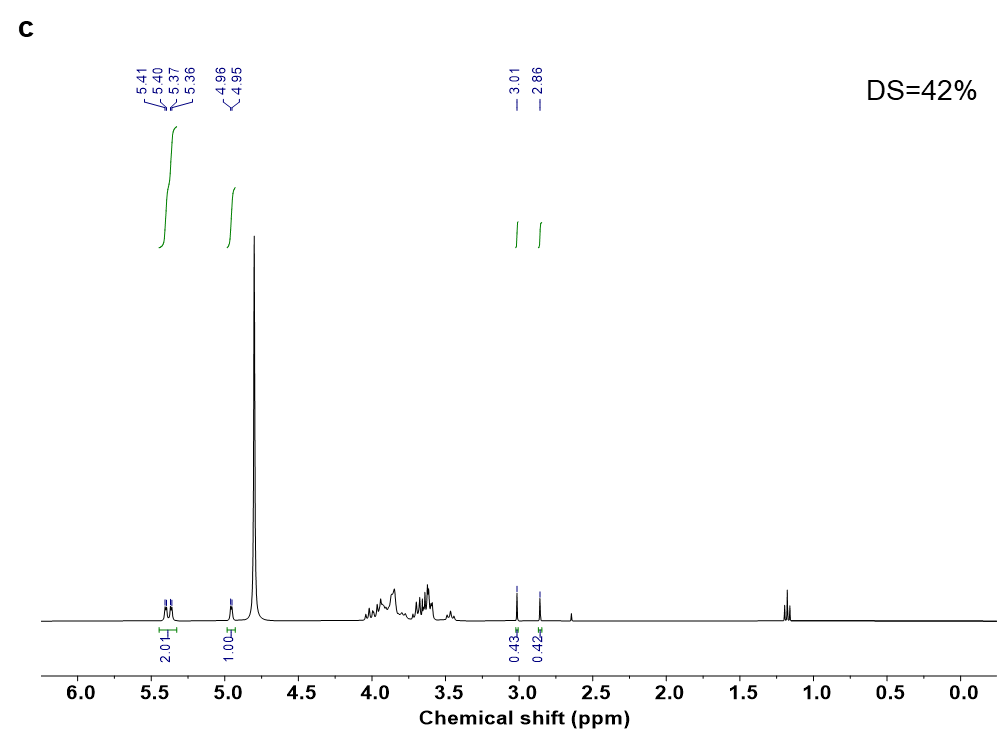
**

**Fig. S1.** ^1^H NMR spectra (500 MHz, D₂O) of pullulan displaying varied degrees of substitution (DS): (a) DS = 9%, (b) DS = 10%, (c) DS = 42%. Characteristic proton assignments are annotated; peak integrals scale proportionally with DS. The characteristic peak a at 5.36-5.41 ppm is assigned to the methylene protons of the repeat unit. The methylene protons of the 1,6-glycosidic bond correspond to the singlet at 4.95 ppm. The methylene protons of the grafted succinic acid moiety give rise to the peaks c at 2.86 and 3.02 ppm. The DS was calculated from the integral ratio of peaks c to peak b.


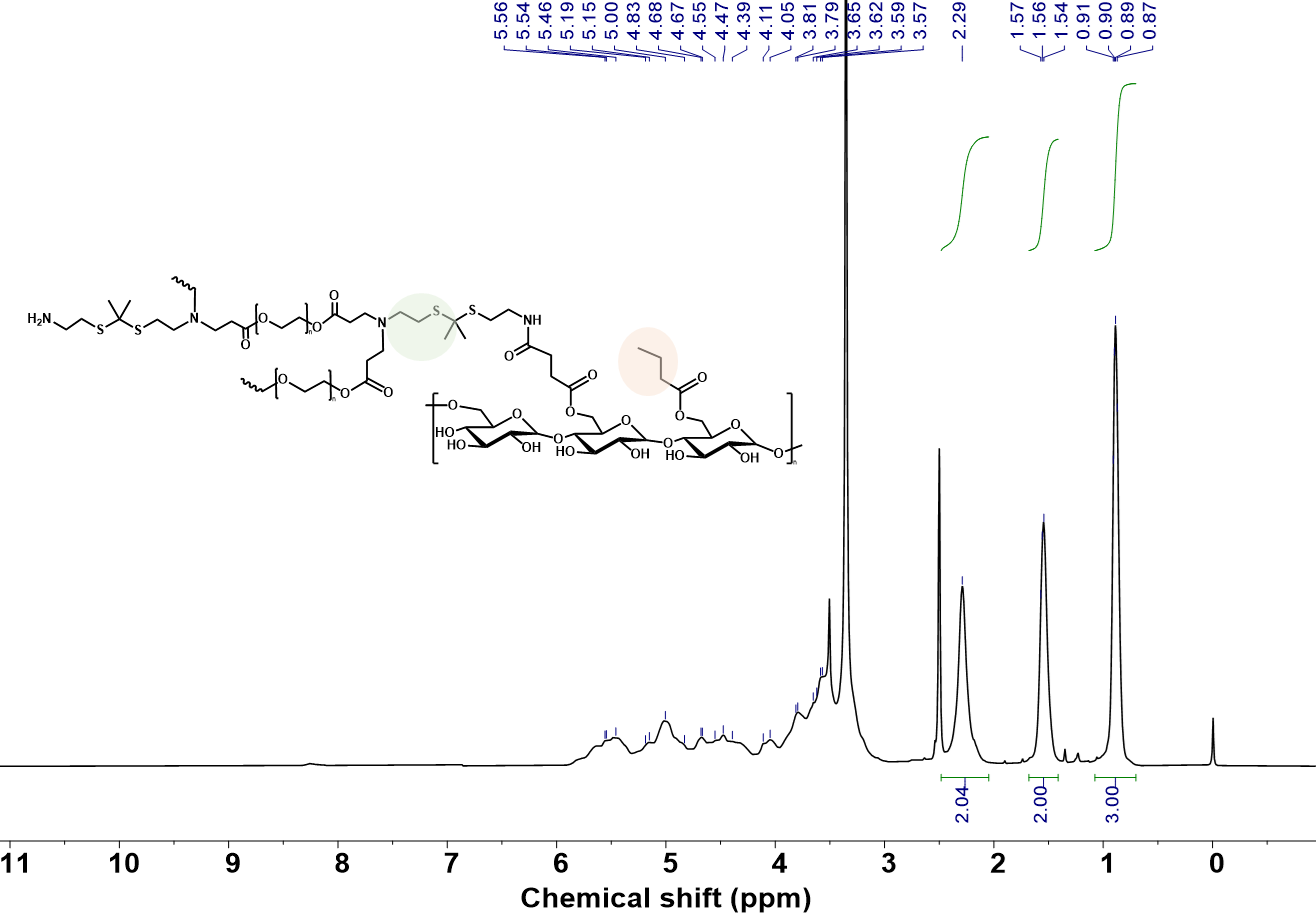


**Fig. S2.** ^1^H NMR spectra (500 MHz, (CD_3_)_2_SO) of PBHB. The protons in both the orange and green regions contribute to the peaks at 2.29 ppm, 1.54-1.57 ppm, and 0.87-0.91 ppm.


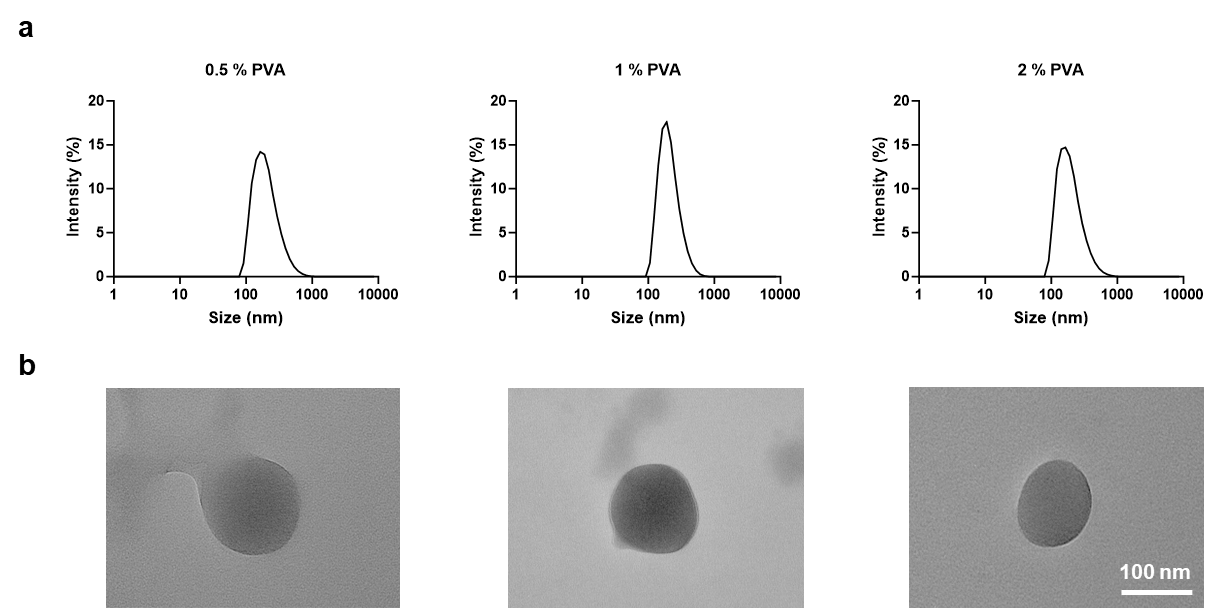


**Fig. S3.** (a) Hydrodynamic diameter of PBHB nanoparticles at varying PVA contents in the aqueous phase and (b) their corresponding TEM images.


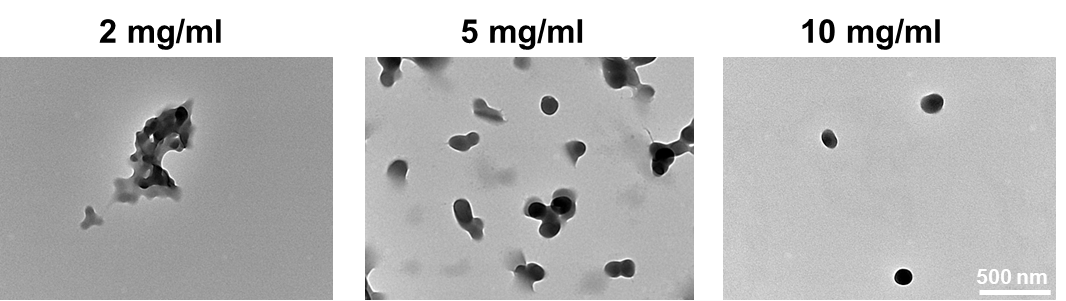


**Fig. S4.** Hydrodynamic diameter of nanoparticles at varying PBHB contents in the organic phase.


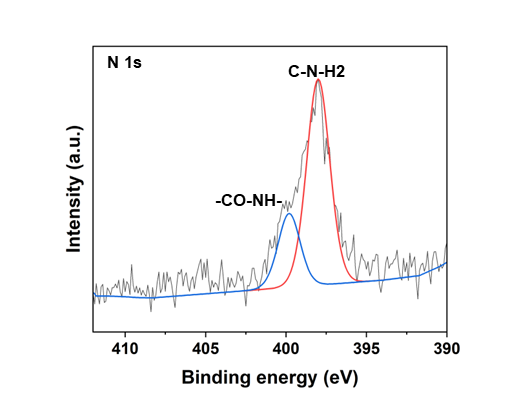


**Fig. S5.** N 1s spectrum of PBHB-based nanoparticles. C–N–H2 represents the C–N bond in a primary amine, while -CO–NH– represents the C–N bond in an amide group[1].

**
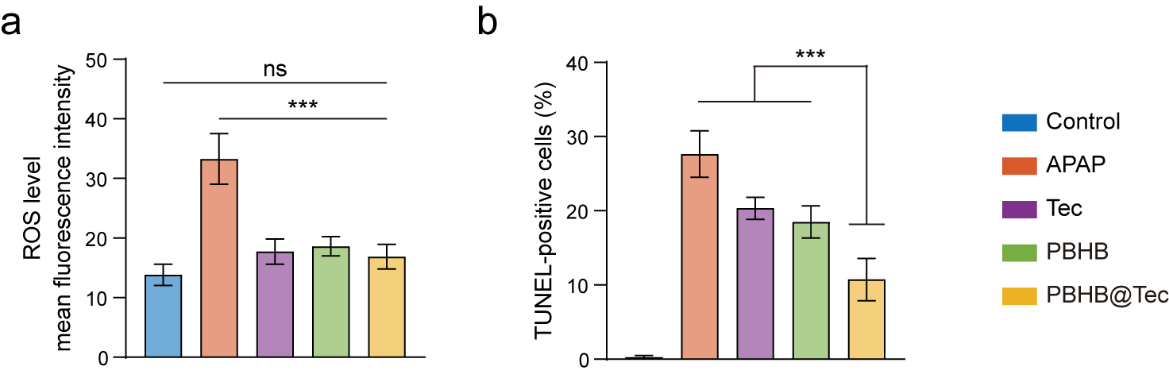
**

**Fig. S6.** (a) Quantification of MFI of DHE staining. (b) Quantification of TUNEL-positive cells. Data are expressed as mean ± SD (n = 6). *p < 0.05; **p < 0.01; ***p < 0.001; ns, not significant.

**
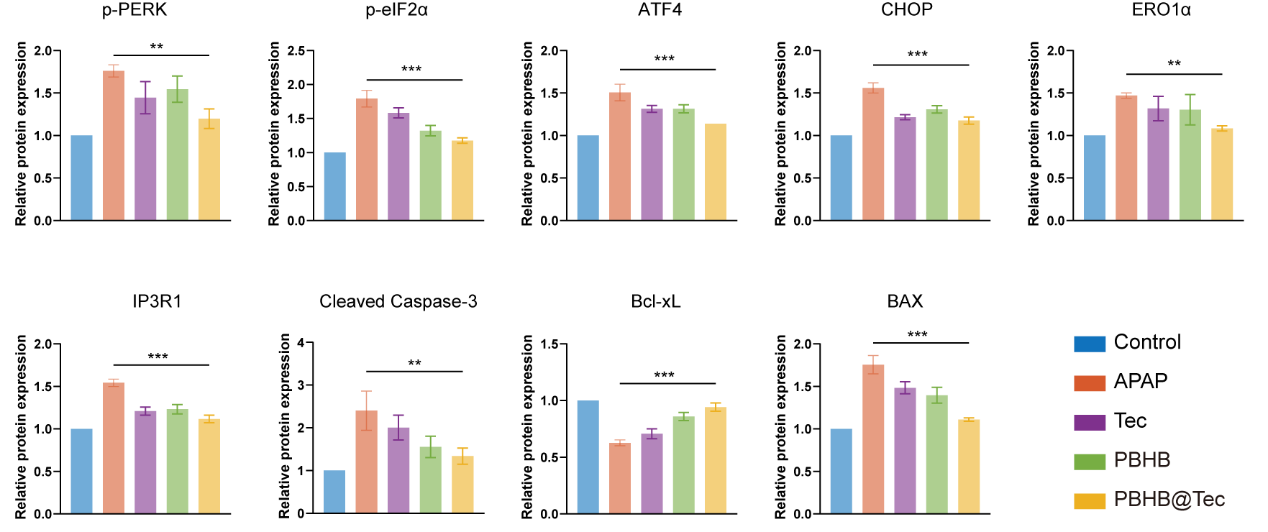
**

**Fig. S7.** Quantification of Western blot analysis of apoptosis- and ER stress-related proteins in mouse liver tissues. Data are expressed as mean ± SD (n = 3). **p* < 0.05, ***p* < 0.01, ****p* < 0.001.

**
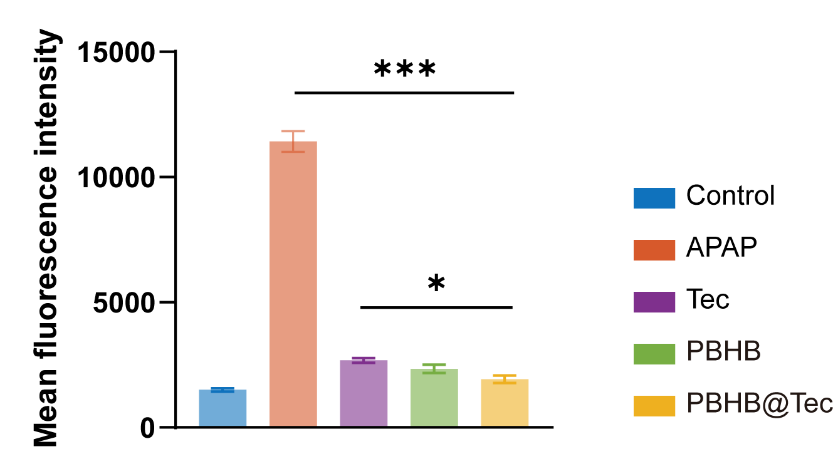
**

**Fig. S8.** Quantitative analysis of mean fluorescence intensity of Rhod-2 AM. Data are expressed as mean ± SD (n = 3). **p* < 0.05, ***p* < 0.01, ****p* < 0.001.

**
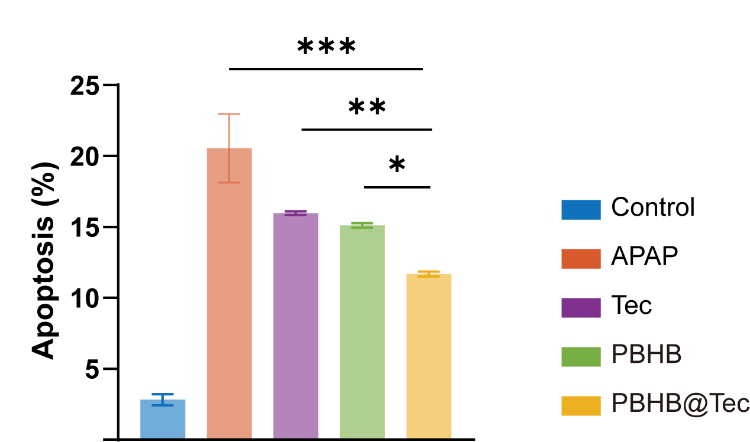
**

**Fig. S9.** Quantification of the percentage of apoptotic cells. Data are expressed as mean ± SD (n = 3). **p* < 0.05, ***p* < 0.01, ****p* < 0.001.

**Fig. S10.** Quantification of Cy5-labeled PBHB uptake by HepG_2_ cells and RAW 264.7 macrophages at different time points as determined by flow cytometry. Data are shown as mean ± SD (n = 3).

**
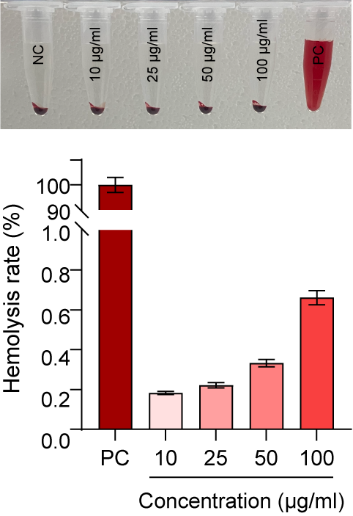
**

**Fig. S11.** Representative images and quantitative analysis of red blood cell hemolysis after incubation with PBHB@Tec at various concentrations. NC: negative control (saline), PC: positive control (deionized water). Data are shown as mean ± SD (n = 3).


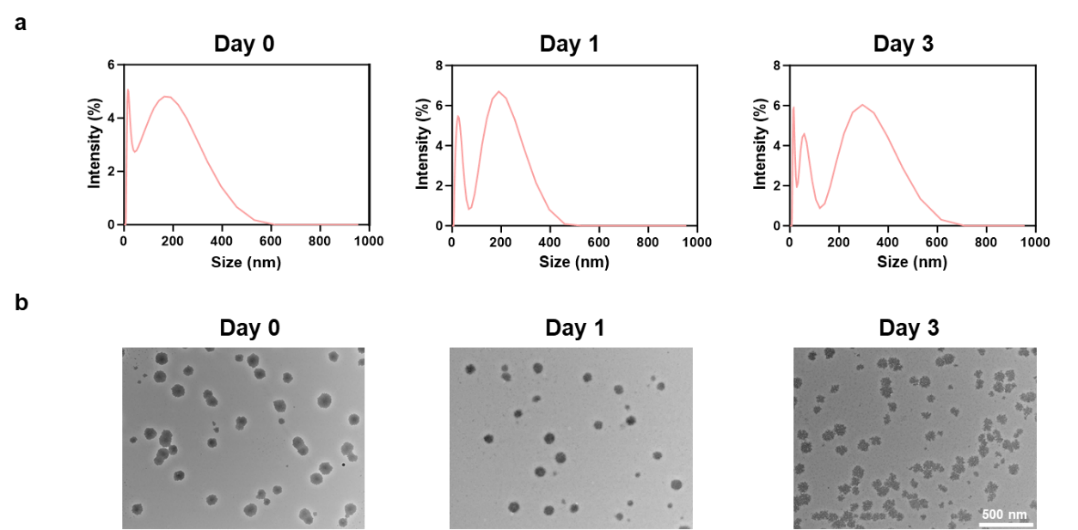


**Fig. S12.** *In vitro* stability of PBHB@Tec. (a) DLS results showing the evolution of the nanoparticles’ hydrodynamic size incubated at 37°C on days 0, 1, and 3. (b) Corresponding TEM images revealing the morphological and dimensional changes of the nanoparticles. At day 0, the sharp peak at the smaller size represents protein aggregates, while the peak at approximately 200 nm corresponds to the nanoparticles. After one day of incubation, the nanoparticle peak showed no significant change—a trend corroborated by TEM images at the corresponding time point. By day 3, however, a new peak emerged between the sizes of the protein aggregates and the nanoparticles. TEM further revealed the formation of small fragments with slight aggregation, suggesting partial nanoparticle degradation.

**
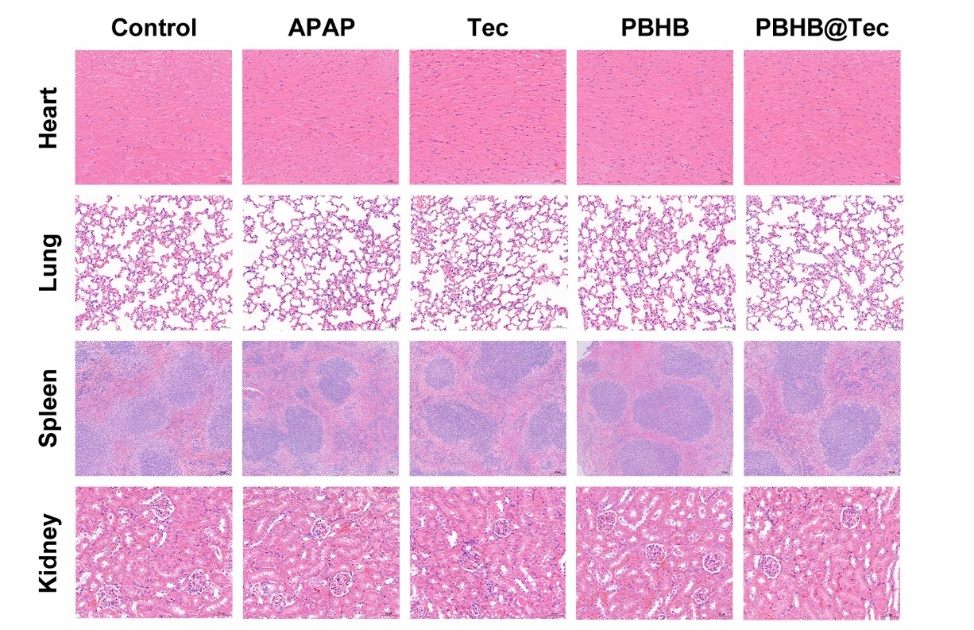
**

**Fig. S13.** Representative H&E staining images of major organs from mice in different treatment groups. No noticeable pathological abnormalities were observed in any group. Scale bar: 50 μm.

**Supplementary Tables**

**Table S1. The sequences of gene primers used for qRT-PCR analysis**

| **Primer** | **Sequence** |
| --- | --- |
| *Actb*-F | CGGTTCCGATGCCCTGAGGCTCTT |
| *Actb*-R | CGTCACACTTCATGATGGAATTGA |
| *Il1b*-F | GAAATGCCACCTTTTGACAGTG |
| *Il1b*-R | TGGATGCTCTCATCAGGACAG |
| *Tnf*-F | CAGGCGGTGCCTATGTCTC |
| *Tnf*-R | CGATCACCCCGAAGTTCAGTAG |
| *Il6*-F | CTGCAAGAGACTTCCATCCAG |
| *Il6*-R | AGTGGTATAGACAGGTCTGTTGG |
| *Il10*-F | CTTACTGACTGGCATGAGGATCA |
| *Il10*-R | GCAGCTCTAGGAGCATGTGG |

**Table S2. Antibodies used for Western blotting**

| **Antibody** | **Source** | **Catalog Number** |
| --- | --- | --- |
| IP3R1 | Cell Signaling Technology | 8568 |
| p-PERK (Ser719) | Proteintech | 29546-1-AP |
| p-eIF2α (Ser51) | Proteintech | 28740-1-AP |
| ATF4 | Cell Signaling Technology | 11815 |
| CHOP | Proteintech | 15204-1-AP |
| ERO1α | Proteintech | 12007-1-AP |
| Cleaved Caspase-3 | Cell Signaling Technology | 9664 |
| Caspase-3 | Abcam | ab184787 |
| Bcl-xL | Cell Signaling Technology | 2764 |
| BAX | Proteintech | 50599-2-Ig |
| β-actin | Cell Signaling Technology | 4970 |
| Anti-rabbit IgG, HRP-linked Antibody | Cell Signaling Technology | 7074 |

**Reference**

[1] M.G. Olayo, E.J. Alvarado, M. González-Torres, L.M. Gómez, G.J. Cruz, Quantifying amines in polymers by XPS, Polym. Bull. 81 (2024) 2319–2328. https://doi.org/10.1007/s00289-023-04829-y.
